# Discovering key transcriptomic regulators in pancreatic ductal adenocarcinoma using Dirichlet process Gaussian mixture model

**DOI:** 10.1038/s41598-021-87234-7

**Published:** 2021-04-12

**Authors:** Sk Md Mosaddek Hossain, Aanzil Akram Halsana, Lutfunnesa Khatun, Sumanta Ray, Anirban Mukhopadhyay

**Affiliations:** 1 Computer Science and Engineering, Aliah University, Kolkata, 700160 India; 2 Computer Science and Engineering, University of Kalyani, Kalyani, 741235 India

**Keywords:** Biomarkers, Computational biology and bioinformatics, Computational models, Machine learning, Statistical methods, Cancer

## Abstract

Pancreatic Ductal Adenocarcinoma (PDAC) is the most lethal type of pancreatic cancer, late detection leading to its therapeutic failure. This study aims to determine the key regulatory genes and their impacts on the disease’s progression, helping the disease’s etiology, which is still mostly unknown. We leverage the landmark advantages of time-series gene expression data of this disease and thereby identified the key regulators that capture the characteristics of gene activity patterns in the cancer progression. We have identified the key gene modules and predicted the functions of top genes from a reconstructed gene association network (GAN). A variation of the partial correlation method is utilized to analyze the GAN, followed by a gene function prediction task. Moreover, we have identified regulators for each target gene by gene regulatory network inference using the dynamical GENIE3 (dynGENIE3) algorithm. The Dirichlet process Gaussian process mixture model and cubic spline regression model (splineTimeR) are employed to identify the key gene modules and differentially expressed genes, respectively. Our analysis demonstrates a panel of key regulators and gene modules that are crucial for PDAC disease progression.

## Introduction

In genetics, gene expression is one of the elementary constitutional blocks which gives rise to a phenotype from a genotype, i.e., a trait which is observable in all living cells, including prokaryotes and eukaryotes. Multiple techniques are available to quantify gene expression and regulation, like DNA microarray, RNASeq, etc. The area of gene expression analysis undergone several significant advancements in biomedical research. With increased efficiency and quality, these measurements led to improvements in disease sub-classification, gene identification problems, and studying progression characteristics of diseases^1–7^. Biological mechanisms are dynamic in nature; therefore, their activities must be supervised at multiple time points. Time-series gene expression experiments are widely used to monitor biological processes in a time-series paradigm^8^. Analyzing these time-series gene expression data helps identify transient transcriptional changes, temporal patterns, and causal effects of the genes. Time-series gene expression studies can be utilized to predict phenotypic outcomes over a period of time^9^.

DNA microarrays and RNASeq data have been accepted as gold standards for analyzing and measuring gene expressions across different biological circumstances^3,5,10^. A gene is considered differentially expressed (DE) if a statistically significant difference in gene expression levels is observed between a pair of experimental conditions. Various statistical distribution models like the Poisson and the Negative Binomial (NB) distribution estimate the differential gene expression patterns. Gene selection refers to detecting the most significant DE genes under various conditions^11^. Selection is made based on a combination of score cutoff, and expression change threshold, commonly generated by the statistical design itself^12^. Popular time-course DE analysis tools include edgeR, DESeq2, TimeSeq, and Next maSigPro based on the NB distribution model. Some DE tools, like ImpulseDE2 and splineTimeR, based on impulse and spline regression models between two groups, respectively, are used on short time-series data^13^.

Gene expression is a strongly regulated spatio-temporal process. Genes having identical expression patterns are associated with the same biological function. Clustering genes with similar expression pattern reduces the transcriptional response complexities by grouping genes responsible for a distinct cellular process^14,15^. Several statistical clustering techniques have been widely used like k-means, hierarchical clustering^16,17^ and self-organizing maps^18^ to produce modules from time-series gene expression profiles. Gradually, various techniques have been developed, especially for clustering time-series data. In^19^, Short Time-series Expression Miner (STEM) has been used as a clustering technique that maps genes to their representative expression profile. Cluster Analysis of Gene Expression Dynamics (CAGED), a clustering technique proposed by *Ramoni et al.*^20^, uses the Bayesian method to model gene-expression dynamics using auto-regressive equations. TimeClust^21^ uses temporal gene expression profiles to produce clusters. TMixClust^22^, Dirichlet process Gaussian process mixture model^14^ are some of the significant non-parametric model-based clustering methods.

The analysis of time-series gene expression modules helped us unravel major biological complications. It provides deep insights into the disease progression^23^, biomarker discovery^24^, identification of hub genes^25^, cell cycle progression^26^, cancer classification^27^ and several other bio-medically important processes. Moreover, the advancements in information system infrastructure facilitated utilizing time-series models more feasible for studying complex psychological phenomena. Numerous tools are now available for enriched network and pathway analysis of the gene modules, enabling further analysis and a deeper understanding of the biological mechanisms.

This article proposed a framework to discover key transcriptomic regulators and key modules from time-series microarray gene expression data in pancreatic ductal adenocarcinoma (PDAC). Initially, differentially expressed (DE) genes were identified by analyzing the empirical Bayes statistics on multivariate time-course gene expression data of PDAC using the splineTimeR^28^. The top 100 DE genes at each time point were analyzed using a R/Bioconductor package Linear Models for Microarray Data (limma)^29^. Dirichlet process Gaussian process mixture model, a non-parametric model-based clustering method, was applied on the DE gene expression profiles to discover gene modules based on the similar responses across the time points^14^. REGulator-Gene Association Enrichment (REGGAE)^30^ was used to identify key transcriptional regulators and the number of targets for each of them from the list of DE genes.

Most experimental gene expression analyses only focus on determining the DE genes by considering them independent events and not investigating the identified genes’ interaction. Reconstruction of the possible gene association network (GAN) among DE genes helps us find genes in the studied phenotype interaction network. Therefore, GAN reconstruction, followed by identifying the top genes in the network, was also performed. Moreover, we have identified regulators for each target gene by gene regulatory network inference for the whole set of genes using the dynamical GENIE3 (dynGENIE3) algorithm^31^ from the time-course gene expression data of PDAC. Prediction of gene functions of the top genes in the interaction network also been carried out using GeneMANIA prediction server^32^. We have identified the key gene modules from the set of all modules obtained from our cluster analysis. Subsequently, transcriptomic regulatory genes were also detected against a curated database of DNA-binding RNA polymerase II TF (DbTF) using TFcheckpoint^33^. Furthermore, biological significance like the Kyoto Encyclopedia of Genes and Genomes (KEGG) pathway^34^, Gene Ontology (GO) and gene-disease associations of the gene modules were also observed using Enrichr^35^.

## Results and discussion

This section provides insight into the detailed findings of our present work.

### Evaluation of differential expression

We have processed normalized gene expression values of 42412 genes described in section "Data preparation". Differential gene expression analysis of the genes among the control and treated samples has been performed using the splineTimeR^28^ described in section "Differential expression analysis". We obtained 1397 DE genes using the adjusted *p*-value $\le 0.05$ with the Benjamini-Hochberg (BH)^36^ correction method and optimum degree of freedom $= 4$. Top 20 DE genes from the PDAC dataset with significant expression value changes were ‘Hs.7413’, ‘CYP26B1’, ‘NPPB’, ‘SFRP4’, ‘Hs.562219’, ‘C12ORF46’, ‘SMAD3’, ‘RN7SK’, ‘RARRES1’, ‘ITGA4’, ‘CLSTN2’, ‘DKK1’, ‘CYP26A1’, ‘EPDR1’, ‘RARB’, ‘LOC340598’, ‘TMEM16C’, ‘INMT’, ‘ACTC1’, ‘C21ORF7’. Supplementary Fig. 1 represents the box, and the violin plot of the cubic spline normalized gene expressions of samples in each condition across each time point for the DE genes.

**Figure 1:**
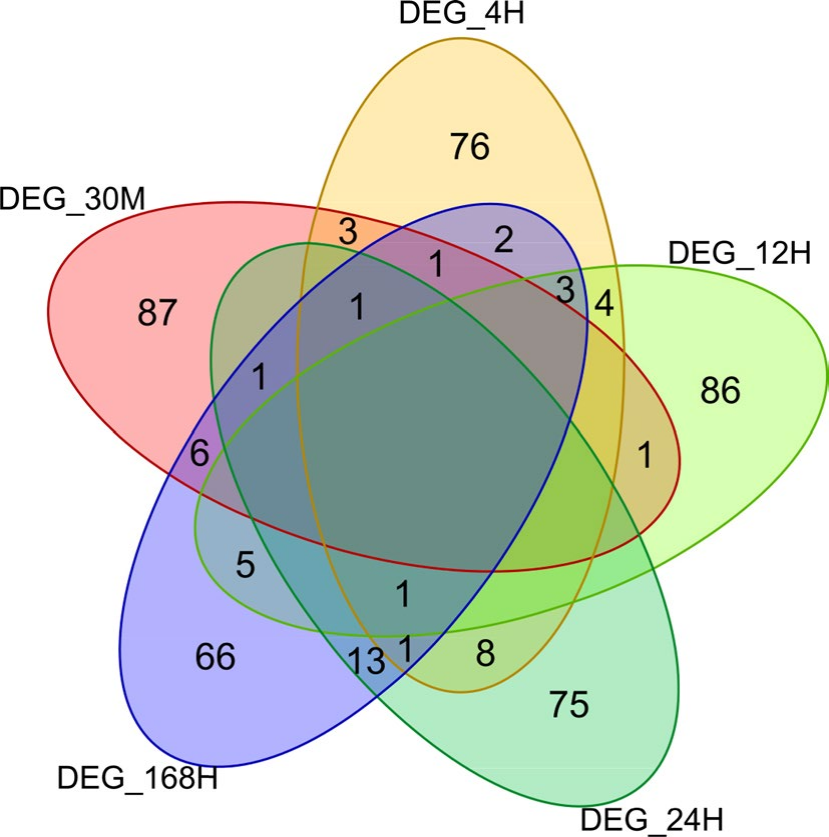
The figure shows the Venn diagram of the number of DE Genes at each time point.

Additionally, to identify the DE genes at each time point, we have employed the limma package^37^. Limma utilizes empirical Bayes smoothing on the estimated fold-changes and standard errors from a linear model fitting. Table 4 shows the top 5 DE genes at each time point, including the top 5 DE genes across all the time points discovered through limma. Figure 1 shows the overlap among the top 100 DE genes between control and treated samples at each time point through a Venn diagram. It has been observed that the top 100 DE genes across all the time points and 168 hours have the highest overlap of 59%, followed by 41% at 24 hours. It was also noticed that the DE genes obtained from the splineTimeR and the top 1397 genes across all the time points through the limma were precisely the same.

### Gene network reconstruction and gene function prediction

Adopting the methodologies discussed in section "Gene association network reconstruction and prediction of gene function", we have reconstructed the gene association network (GAN). We have obtained two GAN’s, each with a different probability cutoff. We discovered 45550 significant edges with 1107 nodes for cutoff probability 0.8 and 34080 significant edges with 1048 nodes for cutoff probability 0.9, which corresponded to 4.67% and 3.5% of all possible edges, respectively. Supplementary Fig. 2 depicts the reconstructed GAN with a probability cutoff set to 0.9 with the top 150 selected edges based on a higher partial correlation score. Genes with higher degrees in the whole reconstructed GAN are displayed in dark colour. From this figure, it can be observed that ‘CUEDC1’, ‘ABCA6’, ‘MRPL50’, ‘LYPD3’, ‘KRT19’, ‘OLFML3’, ‘LGALS3’, ‘Hs.540914’, ‘LOC285074’, ‘TBPL1’ are the top 10 genes having extremely high connection with others within the GAN. Reconstructed GANs highlighting the betweenness and closeness centralities for the top 150 selected edges based on higher partial correlation scores are shown in Supplementary Fig. 3–4. It was discovered that ‘CUEDC1’, ‘ABCA6’, ‘MRPL50’, ‘LYPD3’, ‘KRT19’, ‘OLFML3’, ‘TBPL1’ were the seven common genes that rank within the top ten using all of these three centrality measures.

**Figure 2:**
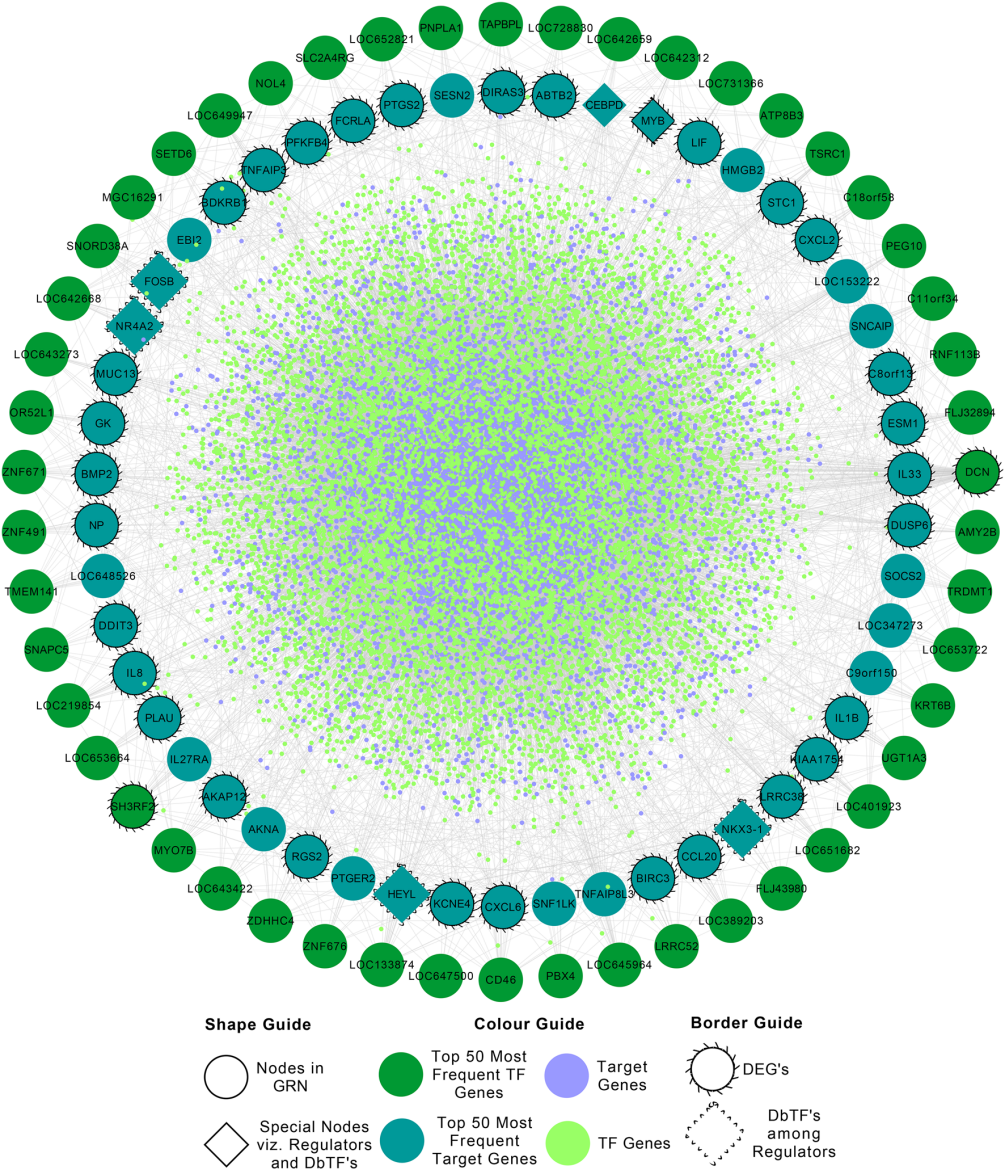
The figure shows the regulatory interactions between transcription factors and target genes in PDAC using dynGENIE3 algorithm.

We have also inferred a gene regulatory network (GRN) using the time-course gene expression data of control and treated samples in PDAC and identified the regulators for each target gene using the dynamical GENIE3 (dynGENIE3) algorithm^31^ as described in section "Gene association network reconstruction and prediction of gene function". Table 2 shows the top 100 regulators for the DE genes (column E). Figure 2 shows the interaction between the regulators and the target genes in the discovered GRN. This figure highlights the top 50 most frequent regulators and 50 most frequent target genes. Supplementary Table 4 reports the interactions among the regulators and the target genes of the inferred GRN with top scores. We have also detected 37 regulators for the 21 targets DE genes with a highly significant interaction score within the whole GRN, reported in Supplementary Table 1. The connectivity patterns of the predicted GRN was also analyzed by enumerating the number of transcription factors (TFs) regulated by a target gene (in-degree) and the number of target genes regulated by a TF (out-degree) [Supplementary Fig. 15]. It has been discovered that most of the TFs regulate a comparatively small number of target genes (low degree TFs), while few TFs regulates a large number of target genes (high degree TFs).

**Figure 3:**
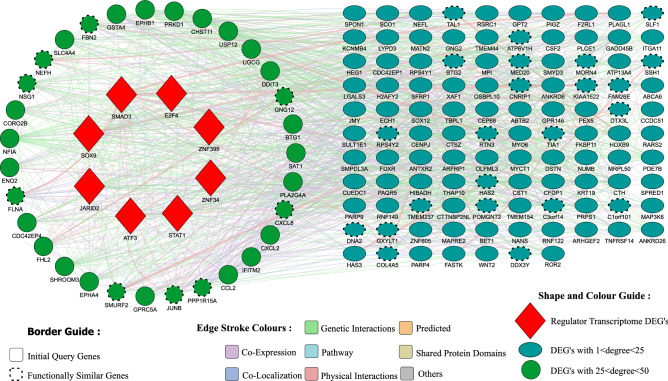
Gene network obtained from the GeneMANIA web server through the Cytoscape software^39^ (version 3.8.2).

We have also extracted the top 150 genes from the reconstructed GAN with 0.9 as the probability cutoff for predicting their gene function, chosen based on the aggregation of three centrality measures: degree, betweenness, and closeness centrality. The top 15 genes among them include ‘CUEDC1’, ‘ABCA6’, ‘MRPL50’, ‘LYPD3’, ‘KRT19’, ‘OLFML3’, ‘LGALS3’, ‘TBPL1’, ‘LOC285074’, ‘ANKRD26’, ‘FDXR’, ‘Hs.540914’, ‘CCL2’, ‘CENPJ’, and ‘CXCL2’. We utilized this list of 150 genes to obtain their gene function using GeneMANIA^32^. GeneMANIA algorithm creates a weighted connected network of the query genes, including several suggested genes. The resultant network is demonstrated in Fig. 3. Table  1 provides the details of the top 5 functions of the resultant genes predicted by the GeneMANIA.

**Table 1:** GeneMANIA predicted functions.

	Function	FDR	Coverage
1.	Cellular response to tumor necrosis factor	0.0015	8/74
2.	Response to tumor necrosis factor	0.0027	8/87
3.	Response to transforming growth factor beta	0.025	9/169
4.	Cellular response to transforming growth factor beta stimulus	0.025	9/169
5.	Cellular response to interleukin-1	0.026	5/36

In Table 1, the coverage column defines the ratio of the number of annotated genes in the resultant network to the number of genes with that annotation in the genome, and FDR is the false discovery rate generated from the GeneMANIA algorithm. The top 50 genes have been chosen from the resultant ranked genes list provided by GeneMANIA with the assigned score for finding their gene-disease associations. It has been observed that ‘CSF2’, ‘HOXB9’, ‘ITGA11’, ‘NUMB’ genes are associated with Pancreatic Ductal Adenocarcinoma according to DisGeNET^38^ web server.

### Transcriptional regulators and DNA-binding transcription factor identification

We have identified the key transcriptional regulators (TR) for the DE genes using REGGAE^30^, with the time-course genes expression profiles of the control and the treated samples. We obtained a ranked list of 66 key TR for the PDAC dataset from a Regulator Target Interactions (RTI) collection, which contains a list of regulators for each deregulated gene that influence gene expression. Regulatory genes were ranked according to the score provided by the REGGAE algorithm. The top 5 key TR’s are ‘GATA6’, ‘NFYB’, ‘IRF1’, ‘TRIM22’, ‘SREBF1’.

Additionally, we have detected the proteins playing central role in DNA transcription using a curated database of specific DbTFs: TFcheckpoint^33^ from the list of key TRs. We retrieved 47 DNA-binding RNA polymerase II TFs (DbTFs) among the 66 key TR’s. Among these DbTFs, we have discovered 10 proteins, viz., ‘FOXO1’, ‘SOX9’, ‘GATA6’, ‘SMAD3’, ‘NFKB1’, ‘KLF6’, ‘TBX3’, ‘SREBF1’, ‘NR4A2’, ‘TCF3’ that are directly associated with PDAC using DisGeNET. We have also observed that among the top 50 ranked genes obtained from the GeneMANIA, there are 8 DNA-binding TF’s: ‘SMAD3’, ‘STAT1’, ‘ZNF34’, ‘ATF3’, ‘ZNF395’, ‘JARID2’, ‘E2F4’, ‘SOX9’.

To validate the identified transcriptional regulators, we have performed an external validation. We first obtain a set of transcription factors from two independent study and then compute the overlap of the predicted set with it. Particularly, we combine transcription factors specific to distinct PDAC subtypes from Giuseppe et al.^40^, with an open source manually curated database of eukaryotic transcription factors called TRANSFAC^41^. We observed that 44 transcription factors of our potential set discovered from REGGAE (containing 66 transcription factors) are common with the combined set (see Supplementary Table 2 for the results). Moreover we observed that our potential set contains three transcription factors ‘SMAD6’, ‘FOSB’ and ‘IRF1’ which are also demonstrated to be important regulators in human pancreatic ductal adenocarcinoma (PDAC)^40^.

### Gene modules identification and determination of key gene modules

**Figure 4:**
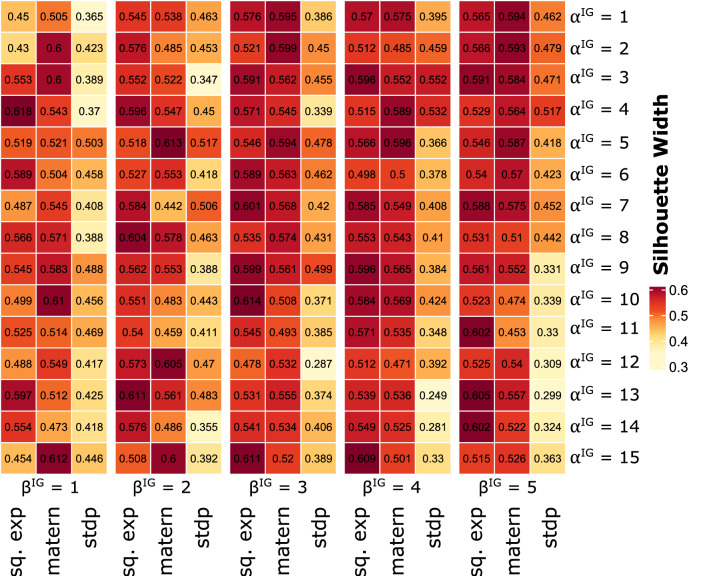
The figure shows the hyperparameter tuning for selecting the best kernel parameters.

After discovering DE genes, we have applied the Dirichlet Process Gaussian Process mixture model to obtain robust and accurate gene modules from their time-course gene expression profiles. We have tuned the hyperparameter of DPGP and uses several kernel functions choices to pick the best model that results in the best clustering solutions. According to a cluster-specific GP, the probability distribution of each gene’s trajectories is defined by a positive definite Gram matrix that quantifies the similarity between every time points. This Gram matrix can be modelled through various kernel functions that depict smoothness and periodicity for GP models. In this work, we have utilized three different kernel functions *squared exponential*^14^, *Matérn52*^42^, and *standard periodic*^43^. We have discovered that the squared exponential (sq. exp) kernel with MAP clustering and Limited-Memory Broyden-Fletcher-Goldfarb-Shanno (L-BFGS) hyperprior optimization technique, concentration parameter ($\alpha = 1.0$), shape ($\alpha ^{IG} = 4$) and rate ($\beta ^{IG} = 1$) parameters for the inverse gamma prior on the cluster noise variance produces best clustering solution with 10 gene modules which yield the highest silhouette width [Fig. 4 and Supplementary Table 3(A–F)]. Additionally, we have compared several other clustering techniques with the current method. We observed that the current method outperforms the other clustering results with respect to the silhouette width in all cases [Supplementary Table 3(G)].

**Figure 5:**
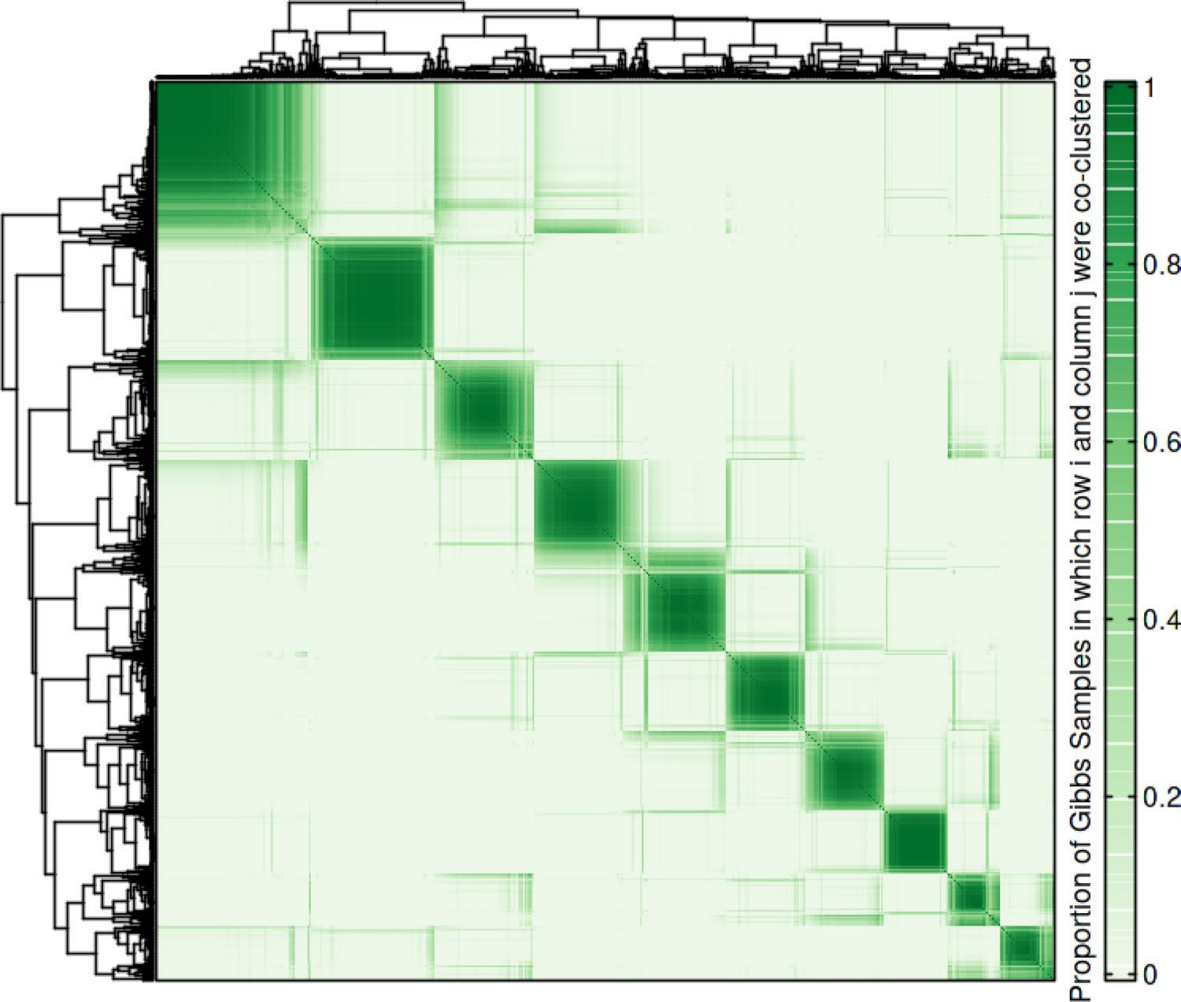
The heatmap shows the posterior similarity matrix obtained from clustering.

**Figure 6:**
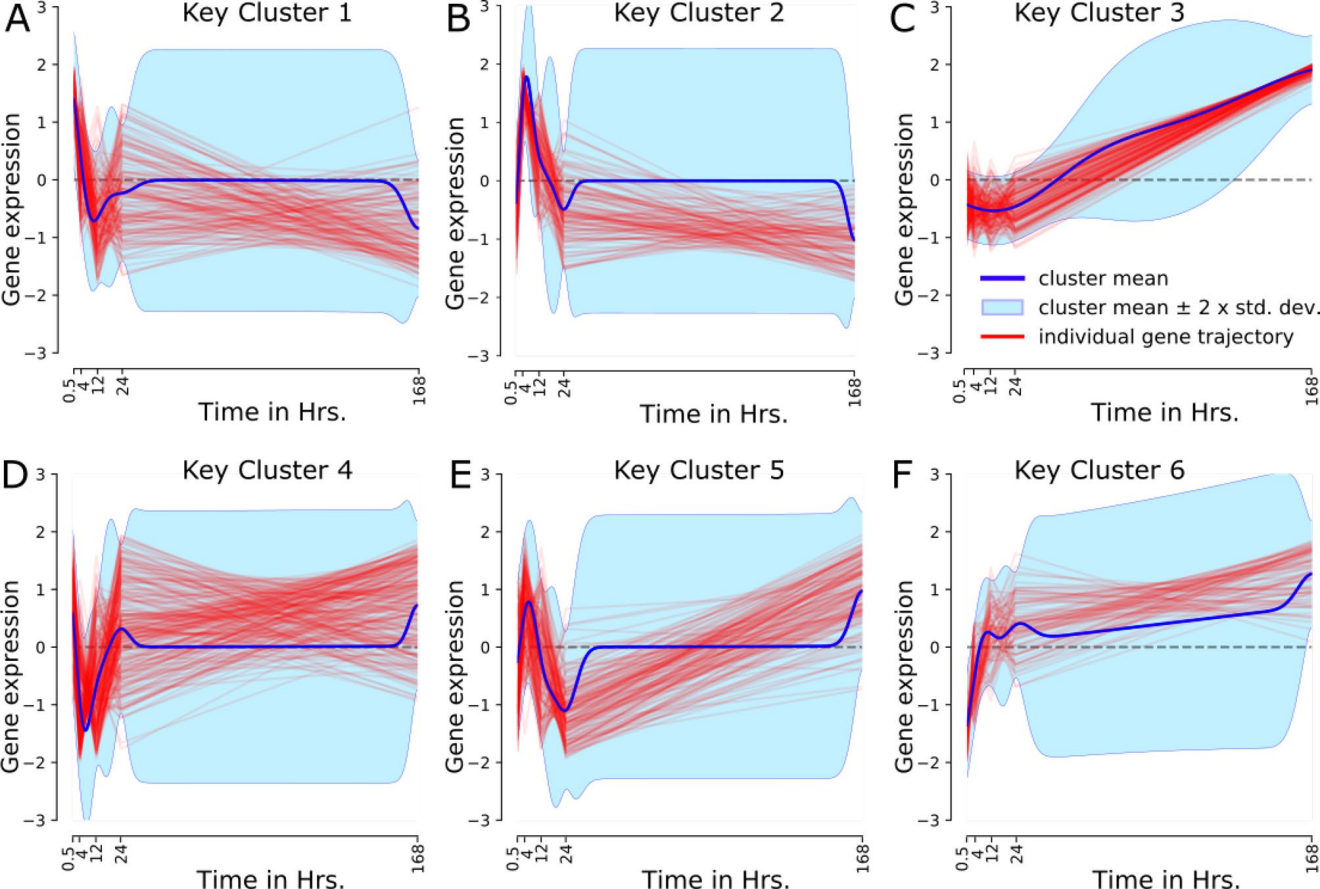
The figure shows the trajectories of gene expression of the DE genes in top six key modules (clusters).

Figure 5 shows the heatmap denoting the posterior distribution of the probability that two genes are being co-clustered. Subsequently, we have selected the top 6 modules with the highest number of regulators and referred to them as the key modules. Figure 6 shows the cluster trajectories of the DE genes for each key module in (A–F), normalized $\log _2$ fold change in expression for each transcript, the posterior cluster mean and $\pm 2$ standard deviations according to the cluster-specific GP.

### Biological significance analysis

**Figure 7:**
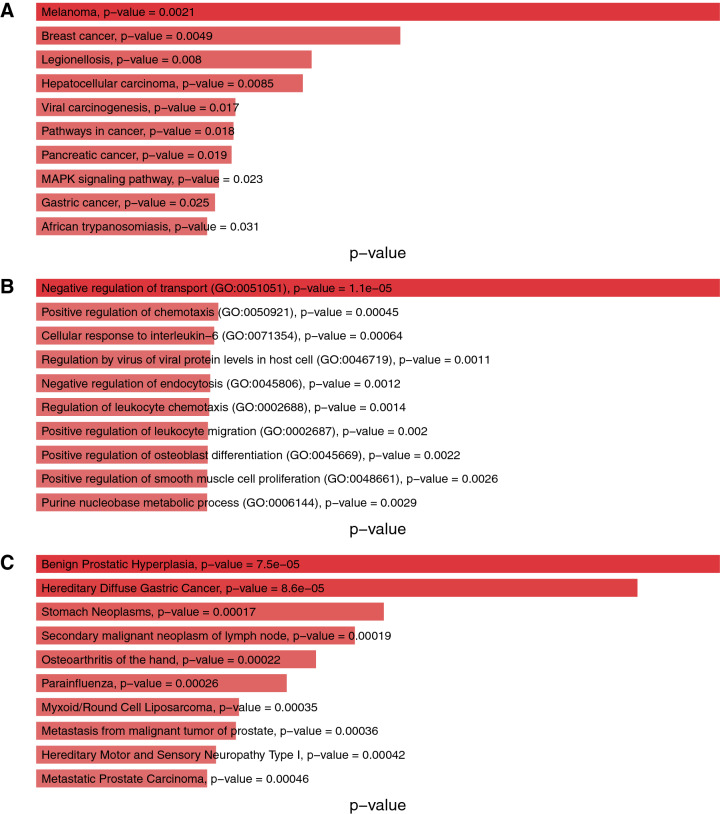
The figure shows the KEGG Pathway (**A**), Biological Processes (**B**), and DisGeNET analysis (**C**) of the key module 1.

We have performed several analysis on the key modules to gain more insights into the pathways and biological processes (BP) of the involved genes inside the key modules. Additionally, we have identified key TRs in each of the key modules. We have identified KEGG pathways, associated biological processes (BP), and disease-gene associations using the DisGeNET database via Enrichr^35^. We have reported our findings, with the name of the regulator genes and DbTF in Table 2, where genes in boldface have been used to represent DbTFs among the TRs. Figure 7 shows the top-ranked (based on *p*-value) KEGG pathways (Panel A), biological processes (Panel B) and disease-genes association (Panel C) enrichment of genes of key cluster 1. Outcomes of our biological significance analysis for the other 5 key modules have been attached in Supplementary Fig. 5–9.

**Table 2:** Biological significance analysis of key regulators in each key module.

**Regulators from REGGAE and DbTF (in bold)**	**Key Cluster**	**Gene Ontology (Biological Processes)**	**Biological Pathway (KEGG)**	**Top 100 GRN inferred regulators**
**GATA6**, NFYB, **IRF1**, TRIM22, **SREBF1**, **RFX5**, PEX2, **HOXA4**, **SMAD3**, ID3, **STAT1**, **FOXO1**, **ZNF34**, RING1, LYL1, TOP2B, **ATF3**, **NR1H4**, **EGR2**, **HOXA5**, **HOXA2**, **RARB**, **HOXC13**, **DACH1**, **ARNT2**, **SMAD6**, TAF15, **FOXF1**, **RELB**, **ZNF395**, **FOSB**, **ZNF281**, ID1, LIN54, **CREB3L2**, **NKX3-1**, **MSX1**, **JARID2**, **TCF3**, **NR4A2**, **RREB1**, **HES4**, **HIVEP1**, **NFKB1**, NCAPG, **ZBTB20**, NOTCH1, **E2F4**, **KLF6**, **RBPJ**, LMNA, **EGR1**, DNMT1, **MYB**, HEXIM1, ZNF280D, ELL2, **PBX3**, **E2F7**, **POU2F2**, **KLF13**, **SOX9**, **TBX3**, **FOSL1**, PRPF4, ZNF792	**Key Cluster 1:** STAT1, ZNF34, ATF3, EGR2, TAF15, FOSB, ZNF281, HIVEP1, ZBTB20, KLF6, PBX3	Negative regulation of transport (GO:0051051); Positive regulation of chemotaxis (GO:0050921)	Melanoma; Breast cancer	SPRR3, OSBP2, TMEM55A, TRAF2, CXCR6, CCDC51, EHD4, NODAL, C5orf37, PLXNA4B, KCTD3, OPTC, LOC645576, RDH10, LOC493869, CIB1, POLA2, LOC645262, RND2, FGFR1OP2, LOC402110, METRN, LOC645681, ATP10B, EAF1, FLJ40712, CXCR7, LOC441052, ANKRD1, UNQ6975, DMRTB1, TMCO3, MYO1B, LOC645451, PRSSL1, CCDC124, POLR3C, NFKBIA, PSMD13, LOC647843, CYB5A, DUSP21, ANKRD31, RUFY1, TMPRSS12, CLEC14A, LOC648603, TRAPPC6B, LOC653554, RUSC2, CCDC115, IVD, C7orf13, MRPL10, L1CAM, YTHDF3, PRLH, LOC643809, LOC644325, DLG3, AVIL, FLJ10781, DDHD1, HS6ST2, PQLC2, OR52N1, LOC653342, FLJ42418, LOC646447, EIF3S6IP, ENDOGL1, ELAVL4, SNORD54, PCBP3, NET1, TMEM61, RGS9BP, LOC652216, FLJ31438, CD58, LOC442582, LOC650632, DDHD2, LOC285307, SMCR7L, LOC644403, SLC17A2, LOC441098, KIAA2026, RHCE, MUC17, LOC389669, FLJ31875, LOC650843, OR5AU1, LOC647058, OR10S1, C17orf39, RPL10L, ATOH1
	**Key Cluster 2:** FOXF1, NKX3-1, MSX1, HES4, NOTCH1, POU2F2, KLF13, TBX3	Artery morphogenesis (GO:0048844); Positive regulation of JAK-STAT cascade (GO:0046427)	IL-17 signaling pathway; TNF signaling pathway	
	**Key Cluster 3:** NFYB, HOXA4, NR1H4, HOXA2, RARB, DACH1, TCF3	Cell projection assembly (GO:0030031); Muscle contraction (GO:0006936)	Focal adhesion; Leukocyte transendothelial migration	
	**Key Cluster 4:** TRIM22, RFX5, ID3, HOXC13, ARNT2, ZNF395, LIN54	Positive regulation of autophagy (GO:0010508); Intrinsic apoptotic signaling pathway in response to endoplasmic reticulum stress (GO:0070059)	p53 signaling pathway; Glycine, serine and threonine metabolism	
	**Key Cluster 5:** LYL1, RELB, JARID2, NR4A2, NFKB1, RBPJ, ZNF792	I-$\kappa$B kinase/NF-$\kappa$B signaling (GO:0007249); positive regulation of cytokine biosynthetic process (GO:0042108)	MAPK signaling pathway; Toxoplasmosis	
	**Key Cluster 6:** GATA6, IRF1, SREBF1, SMAD3, TOP2B, HOXA5	Regulation of transforming growth factor beta2 production (GO:0032909); Negative regulation of blood vessel morphogenesis (GO:2000181)	Adherens junction; Pancreatic cancer

Moreover, we have identified the top 10 KEGG pathways and GO terms (BP) for each of the key modules to understand their relevance and significance in PDAC. We observed that key module 1 contains ‘FGF7’, ‘IL6’, ‘FGF9’, ‘GSTA4’, ‘GADD45A’, ‘CDK4’, ‘STAT1’, ‘TXNRD1’, ‘WNT2’ genes which contributes to pathways in cancer and ‘STAT1’, ‘GADD45A’, ‘CDK4’ are directly associated to pancreatic cancer pathway. ‘IL6’, ‘STAT1’, ‘ST3GAL6’ inside the key module 1 is responsible for cellular response to Interleukin 6 (IL -6) that governs the pancreatic cancer progression^44^. ‘CXCL6’, ‘CSF2’, ‘CCL20’, ‘TNFAIP3’, ‘CXCL1’, ‘PTGS2’ inside key module 2 takes part in IL-17 signaling pathway that promotes the transition to pancreatic cancer from chronic pancreatitis^45^. ‘TNF-$\alpha$’ expression elevates in PDAC initiation process which binds to ‘TNFR1’ receptor resulting in Tumor Necrosis Factor(TNF) signaling^46^. ‘CSF2’, ‘CCL20’, ‘LIF’, ‘TNFAIP3’, ‘CXCL1’, ‘PTGS2’ in key module 2 influences the TNF signalling pathway. Moreover, ‘IL11’, ‘CSF2’, ‘LIF’, ‘SOCS5’ participates in JAK-STAT signaling pathway which directly involves in pancreatic tumorigenesis^47^ and ‘CSF2’, ‘NOTCH1’, ‘CLCF1’, ‘LIF’, ‘CRLF1’ genes are responsible for positive regulation of JAK-STAT cascade which have been detected in the key module 2. ‘LFNG’, ‘NOTCH1’, ‘NUMB’ in key module 2 are actively involved in Notch signaling pathway which is reactivated in PDAC initiation and development^48^. Focal Adhesion Kinase (FAK) play a crucial role and is highly activated and over-expressed in PDAC^49^. In key module 3, we discovered ‘MYL7’, ‘SHC3’, ‘ITGA4’, ‘COL4A4’, ‘ITGA1’, ‘FN1’, ‘FLNA’, ‘MYL9’ that take part in the Focal adhesion pathway. Key module 3 also contains ‘SFRP4’, ‘FOXC1’, ‘NCOA3’ which give rise to protein N-linked glycosylation via asparagine process. Unusual glycosylation has been recognized as a molecular feature of malignant transformation in PDAC^50^. Disease progression in PDAC is impacted by mutant ‘p53’ tumor suppressor^51^. Presence of ‘GADD45B’, ‘SESN1’, ‘TNFRSF10B’, ‘PPM1D’ genes in the key module 4 indicates their involvement with ‘p53’ signaling pathway. ‘GPT2’, ‘GLS’ genes in key module 4 play roles in Arginine biosynthesis. Arginine metabolism is spiked in PDAC cells and it’s deprivation may be considered as a potential strategy for PDAC therapy^52^. Key module 4 contains ‘DDIT3’, ‘TNFRSF10B’, ‘TRIB3’, ‘CHAC1’ genes which are active in intrinsic apoptotic signaling pathway in response to endoplasmic reticulum (ER) stress. ER stress contributes to pancrititis, a risk factor for PDAC^53^. Pancreatic cancer can be recognized by integral activation of the mitogen-activated protein kinase (MAPK) pathway^54^. A large number of genes, ‘CSF1R’, ‘IL1A’, ‘TGFB3’, ‘PDGFC’, ‘PLA2G4C’, ‘HSPB1’, ‘FGF2’, ‘RASGRP1’, ‘NFKB1’, ‘HSPA1B’, ‘RELB’ of the key module 5 are involved in the MAPK signaling pathway. Moreover, ‘NFKBIA’, ‘NFKB1’, ‘BIRC3’, ‘RELB’ inside the key module 5 are involved in the Nuclear Factor-kappaB (NF-$\kappa$B) signaling pathway that play an important role in PDAC progression, development and are frequently found in upregulated PDAC cells^55^. Also, a subset of genes in the key module 5, ‘NFKBIA’, ‘RIPK2’, ‘IRAK2’, ‘NFKB1’, ‘BIRC3’, ‘RELB’, ‘PRKCH’, ‘AKR1B1’, ‘HSPA1B’ are responsible for I-$\kappa$B kinase/NF-$\kappa$B signaling and positive regulation of NF-$\kappa$B transcription factor activity which has vital roles in constitutive growth of PDAC. Interleukin-1 (IL-1) induces activation of NF-$\kappa$B transcription factor^56^. ‘NFKBIA’, ‘IL1A’, ‘RIPK2’, ‘IRAK2’, ‘SQSTM1’, ‘NFKB1’, ‘ANKRD1’ inside the key module 5 play active roles in IL-1-mediated signaling pathway and cellular response to IL-1. High level expression of functional adherens cell junctions has been seen in cultured cells of PDAC (in-vitro)^57^. In key module 6, ‘PTPRB’, ‘SMAD3’, ‘RAC2’ genes are associated with the Adherens junction pathway. We found that ‘TGFB2’, ‘SMAD3’, ‘RAC2’ inside key module 6 are directly associated with Pancreatic cancer pathway. Additionally, ‘TGFB2’, ‘SMAD3’, ‘INHBE’ are responsible for TGF-beta signaling pathway in the key module 6. Activation of the TGF-$\beta$ signaling pathway leads to increased chemotherapeutic resistance of pancreatic cancer cells^58^.

**Figure 8:**
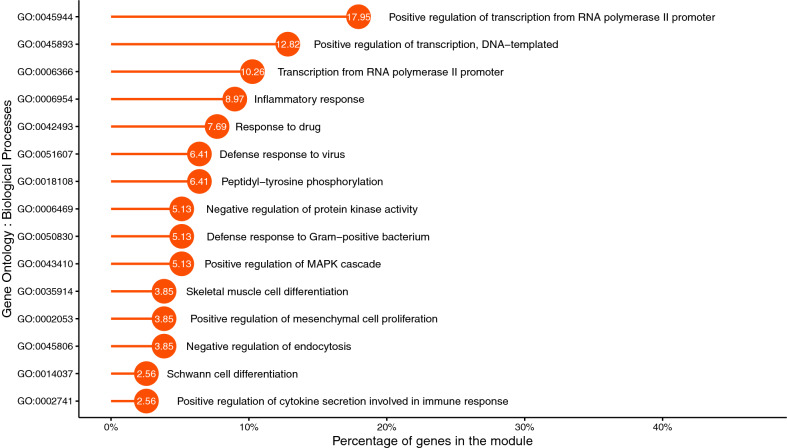
The figure shows the lollipop plot describing the percentage of genes in key module 1, contributing to the top 15 gene ontology terms.

Figure 8 shows the top 15 gene ontology terms (biological processes) performed by the genes in the ‘key module 1’ with their proportion of counts in the module. These GO terms have been selected according to the lowest *p*-values. This analysis for the other 5 key modules has been attached in the Supplementary Fig. 10–14. Additionally, we have analyzed genes in the top 6 key modules for their direct association with PDAC through the DisGeNET web server. We have found that 11, 10, 13, 11, 11, 7 genes are directly associated with PDAC in the key modules 1, 2, 3, 4, 5, 6, respectively. The names of the genes have been tabulated in Table 3.

**Table 3:** Genes related to PDAC in each key module.

Key module 1	Key module 2	Key module 3	Key module 4	Key module 5	Key module 6
IL6, F2RL1, CLIC3, PRKD1, TNFRSF11B, ROR2, CDK4, FGF7, ERBB3, KLF6, ITGA11	PTGS2, LGALS3, CSF2, KRT19, NOTCH1, ISG20, TBX3, NUMB, MTSS1, KHDRBS1	CDKN2A, IGF2BP3, FN1, CXCL12, FOXC1, NCOA3, TCF3, ST6GAL1, SFRP4, CNN2, MMP11, IDH1, RPL17	NUPR1, TNFRSF10B, ADM, SAV1, SLC7A5, KRT7, KIT, GCHFR, DDR1, BTG1, CCDC51	IL1A, TFPI2, HOXB9, NFKB1, THBS1, GDF15, SQSTM1, TAP1, NR4A2, LIMS1, CADM1	GATA6, IGFBP3, SMAD3, SREBF1, TIMP3, GPRC5A, PCDH10

## Methods

**Figure 9:**
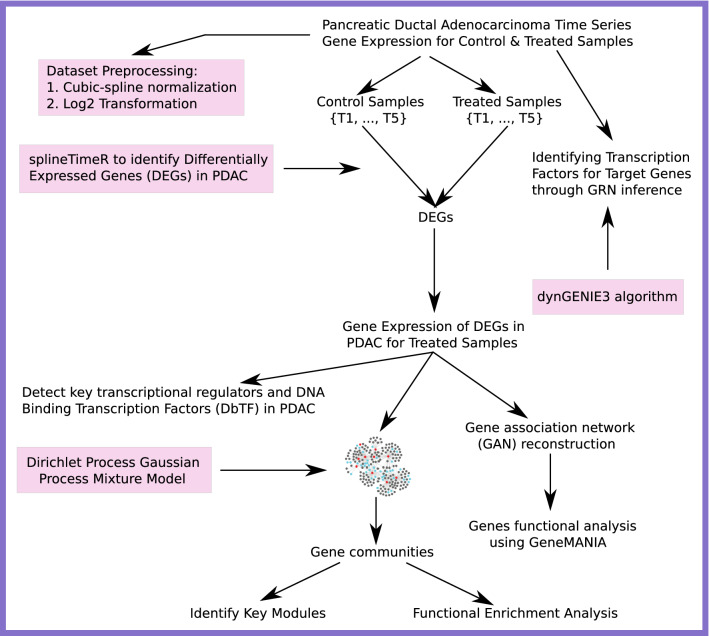
The figure shows an overview of the whole framework adopted in the present work.

The present section provides an overview of our systematic approach to data collection, data preprocessing, and the overall framework for the different methodologies used in our present analysis [Fig. 9].

### Data preparation

We have collected the PDAC microarray gene expression profiles of pancreatic stellate cell line samples from the Gene Expression Omnibus (GEO) via the accession number GSE14426^59^. The dataset has 48701 genes and 30 samples of each gene, along with their periodic gene expression changes. Samples include cubic spline-normalized intensity values of two conditions, viz., pancreatic stellate cell line before and after being treated with a 1-molecular concentration of all-trans retinoic acid (ATRA) on plastic. The gene expressions were recorded at 5-time points (30 min, 4 hours, 12 hours, 24 hours and 168 hours), having 3 replicates at each time point^60^. Gene expression dataset was $log_{2}$-transformed before further analysis. Annotation of Illumina gene identifiers to official gene symbols was carried out by augmenting the information available in the GEO Illumina human platform information (GPL6102) and the R/Bioconductor package *org.Hs.eg.db*^61^. Later, we have used the gene expression profiles of the annotated 42412 official genes for our differential gene expression (DE) analysis.

### Differential expression analysis

Analysis and detection of differential expression of genes have been carried out using the R/Bioconductor *splineTimeR* package^28^ to discover significant DE genes. SplineTimeR operates on the values obtained from the parameters of a fitted natural cubic spline regression model, which is utilized in our time-course gene expression profiles for control and treated samples^28^ (for a detailed description see Supplementary text). The differential expression of a gene has been discovered by applying empirical Bayes moderated F-statistics on the spline regression model’s coefficient differences. The detection of DE genes using splineTimeR^28^ has been carried out by setting the Benjamini-Hochberg^36^ adjusted *p*-value threshold to 0.05 and with a degree of freedom of 4 for all genes. Top regulated genes have been identified as differentially expressed and used for our subsequent analysis in module discovery. Supplementary Fig. 1 represents the box and the violin plot of the cubic spline normalized gene expressions of samples in each condition across each time point for the DE genes.

Additionally, to identify the DE genes at each time point, we have employed the limma package^37^. Limma utilizes empirical Bayes smoothing on the estimated fold-changes and standard errors from a linear model fitting to assesses the differential gene expression across a pair conditions. Table 4 shows the top 5 DE genes at each time point, including the top 5 DE genes across all the time points discovered through limma.

**Table 4:** Differentially Expressed Genes using Limma.

30 minute	4 hour	12 hour	24 hour	168 hour	Final DE Genes
SNAR-A1	CXCL2	FKBP11	CYP26B1	CYP26B1	Hs.7413
HMOX1	CCL20	MYH10	SYNPO2L	FHOD3	CYP26B1
LFNG	DHRS3	CRLF1	CXCL1	NPPB	NPPB
BCYRN1	IL8	SLC12A8	NPPB	ACTC1	SFRP4
LOC255783	SMAD3	Hs.559673	LOC340598	SFRP4	Hs.562219

### Gene network reconstruction and prediction of gene function

#### GAN Construction

In this work, we have performed a gene association network (GAN) reconstruction from the identified DE genes’ time-course data using a regularized dynamic partial correlation method^62^. GeneNet^62^ has been used to analyze covariance matrices with a dynamic shrinkage method. Analyses have been performed with a posterior probability cutoff of 0.8, and 0.9. GAN reconstruction has been implemented using splineNetRecon function from the splineTimeR package^28^ to identify regulatory association between genes under a specific condition (treatment)^28^. Top significant edges have been identified based on their cutoff posterior probability. We have identified the top genes by analyzing the overall graph topology in the resultant GAN based on a higher partial correlation score and widely used centrality measures: degree, betweenness and closeness centrality. These centrality measures were aggregated for selecting the top 150 ranked genes by taking the average of the quantile normalized values of these measures to perform gene function prediction.

GeneMANIA^32^ infers possible connections between the query genes by searching many large, publicly available biological databases to find related genes. These include gene and protein expression data, protein-protein, protein-DNA and genetic interactions, pathways, reactions, protein domains and phenotypic screening profiles. GeneMANIA operates on a ridge regression-based fast heuristic algorithm to integrate multiple functional gene association networks using a label propagation algorithm. GeneMANIA weights data sources based on all genes’ connectivity strength with each other in the query list and suggests relatively similar non-queried genes and their connection types. It returns an interactive functional gene association network of all resultant genes and their relationship. It also performs gene functions prediction of queried genes based on non-negative weights of gene sources (i.e. association of two genes) as a binary classification problem. GeneMANIA further returns a ranked gene list presumably sharing phenotypes with queried genes based on it’s large and diverse databases. We have analyzed the top 150 ranked genes obtained from GAN for gene function prediction using GeneMANIA^32^.

#### GRN Inference

Dynamical GENIE3 (dynGENIE3)^31^ is an adaptation of the GENIE3 method for gene regulatory network (GRN) inference that handles time series and steady-state data jointly. It is based on a semi-parametric model that models gene expression’s temporal evolution using a non-linear ordinary differential equation (ODE). An ensemble of non-parametric regression tree (Random Forest) model is used to learn transcription function in each ODE. It assesses each input feature’s importance to measure the variable importance scores (VIS) by considering the Mean Decrease Impurity measure. Finally, it uses the normalized sum of VIS to assign ranks for each learning sample from which the tree was built to identify regulators of each target gene. In our work, we have identified regulators for each target gene by GRN inference for the whole set of genes using the dynGENIE3 algorithm from the time-course gene expression data of PDAC.

### Finding gene modules from time-series data

Piece-wise Aggregate Approximation (PAA) has been used from the *TSrepr* R package^63^ for dimensionality reduction of multivariate time-series data. The DEGs obtained from the dataset contained 3 replicate values for each time point, which have been converted into univariate expression values using the mean function of PAA. These expression values were clustered to form the gene modules.

We have utilized a non-parametric model-based approach, the Dirichlet Process Gaussian Process mixture model (DPGP), presented by McDowell *et al.*^14^ to obtain gene modules from the univariate time-course expression data. It can simultaneously model cluster number with a Dirichlet process (DP) and temporal dependencies with Gaussian Processes (GP). DPGP uses a Bayesian non-parametric model for time course paths $P \in {\mathbb {R}} ^{N \times T}$, where *N* is the number of genes and *T* is the number of time points (see the Supplementary text for a detailed description of DPGP method). For Markov chain Monte Carlo (MCMC), we have estimated the probability of the trajectory of gene *j* inside the cluster *i* according to the DP prior with the likelihood that gene *j* belongs to class *i* according to the cluster-specific GP distribution. Neal’s Gibbs Sampling “Algorithm 8” has been used to compute the posterior distribution of the trajectory class assignments^64^.

We have executed the MCMC algorithm with two burn-in phases that converged by cluster-switching ratio. After the second burn-in phase, the DPGP updates the model parameters and computes the kernel hyperparameters’ cluster-specific posterior probabilities by the type II maximum likelihood. For this purpose, we have compared the performance of three different optimization techniques, viz., the fast quasi-Newton limited-memory Broyden-Fletcher-Goldfarb-Shanno (L-BFGS), the function minimization using gradient information in a truncated Newton (fmin_tnc), and stochastic conjugate gradient (SCG)^65^. Here, the cluster assignment vector is sampled at every $\hbox {s}^{th}$ iteration ($\hbox {s} = 3$, here) to thin the Markov chain^14^. MCMC generates a sequence of states produced by a Gibbs sampler, where each state delineates a group of genes into disjoint modules. Results generated from Gibbs samples were compiled into a posterior similarity matrix (PSM). The outcomes from the Markov chain was summarized with three different clustering criteria, viz., the maximum a posteriori (MAP), the maximization of posterior expected adjusted rand (MPEAR), and the least-squares^66^ to discover the best cluster assignment. DPGP conservatively declares convergence ensuring convergence plateau for at least 10 samples based on the iterative changes in posterior log-likelihood or the squared distance between sampled partitions and the posterior similarity matrix. We have also performed hyperparameter tuning for finding the concentration parameter ($\alpha$), shape ($\alpha ^{IG}$) and rate ($\beta ^{IG}$) parameters for the best clustering solution, which yields the highest silhouette width.

### Key module identification and biological significance analysis

This subsection provides information about approaches that have been used to find the biological significance of our obtained results.

#### Identification of transcriptional regulators and DNA-binding transcription factors

We have utilized time-course expression profiles of the DE genes across the control and treated samples to identify the key transcriptional regulators (TR) using REGGAE^30^. It operates by using Kolmogorov-Smirnov-like test statistics and an implicit combination of regulator target interactions (RTI’s) for the prioritizing influence of TR’s. REGGAE returns a ranked list of key TRs with p-value aggregations. It uses an extensive collection of RTIs that relies on diverse external databases: ChEA, ChipBase, ChIP-Atlas, ENCODE, Signalink, Jaspar and TRANSFAC. Some of these databases provide manually curated RTIs extracted from the primary literature, while others provide binding information extracted from ChIP-Seq experiments. Additionally, we have also identified regulators for each target genes through the gene regulatory network inference analysis. We have also identified the DNA binding transcription factors (DbTF) through a cumulative and high-quality knowledge source of genome-scale information, TFcheckpoint^33^, from the detected transcriptional regulators obtained using REGGAE. The TFs in TFcheckpoint are investigated for experimental evidence supporting their role in specific DNA binding activity and RNA polymerase II regulation.

#### Functional enrichment analysis of the key modules

Identification and analysis of key gene modules which contain the highest number of transcriptional regulators have been performed. The top 6 key modules have been analyzed to discover biological processes, pathway analysis and disease-gene associations for the involved genes inside each module using Enrichr^35^. Additionally, we have studied the genes inside the key modules for their direct associations with PDAC.

## Conclusion

This article developed a framework to discover the key regulatory genes and the key gene modules from multivariate time-series Pancreatic Ductal Adenocarcinoma (PDAC) microarray data. Here, we have identified the top differentially expressed (DE) genes with a cubic spline regression model. We have performed the Gene Association Network (GAN) reconstruction to discover the regulatory association between genes in PDAC. Moreover, identifying key regulatory genes for each target gene has been carried out through a GRN inference analysis. Additionally, we have detected transcriptional regulators and DNA binding Transcription Factors (DbTFs) using REGulator-Gene Association Enrichment (REGGAE) and TFcheckpoint databases. Gene modules have been identified through a Dirichlet Process Gaussian Process (DPGP) mixture model. We have identified and analyzed the top 6 key gene modules that contain a significant number of regulatory genes. Biological significance analysis reveals that the genes inside the key gene modules are highly associated with PDAC.

Our analysis can be further extended by analyzing integrated multi-omics data of PDAC patients. Common symptoms of PDAC include weight loss, indigestion, abdominal and back pain. Thus, studying pathway networks of the key gene modules may unravel deep insights into this disease. Moreover, the genes inside the key modules may be further validated using *in vitro* experiments to reveal some important findings in PDAC and its pathogenesis. One may also verify the role of key regulators in the modules as potential biomarkers. Survival analysis of the key transcriptional regulators may enlighten us with more significant insights about this disease.

## Supplementary Information

Supplementary Information (pdf 6408 KB)

## Data Availability

We have utilized the following software packages in our present study: Inkscape (version 1.0.2), ComplexHeatmap^67^, ggplot2^68^, venn^69^, Cytoscape^39^ (version 3.8.2) to produce the images.
