# Supplementary material for: Discovering key transcriptomic regulators in pancreatic ductal adenocarcinoma using Dirichlet process Gaussian mixture model
Source: Sci Rep. 2021 Apr 12;11:7853. doi: 10.1038/s41598-021-87234-7 (PMC8041769; doi:10.1038/s41598-021-87234-7)
Supplement: Supplementary file 1 — Supplementary Information (pdf 6408 KB) [file 41598_2021_87234_MOESM1_ESM.pdf]

Title: Discovering Key Transcriptomic Regulators in Pancreatic Ductal Adenocarcinoma using Dirichlet Process Gaussian Mixture Model

Authors:

Sk Md Mosaddek Hossain[1,2,+,*], Aanzil Akram Halsana[1,+], Lutfunnesa Khatun [2], Sumanta Ray[1], and Anirban Mukhopadhyay [2]

[a] Department of Computer Science and Engineering, Aliah University, Kolkata, India, 700160.

[b] Department of Computer Science and Engineering, University of Kalyani, Kalyani, India, 741235.

*mosaddek.hossain@gmail.com

Supplementary text

1 A short description on splineTimeR

Analysis and detection of differential expression of genes have been carried out using the R/Bioconductor *splineTimeR* package to discover significant DE genes. SplineTimeR operates on the values obtained from the parameters of a fitted natural cubic spline regression model, which is fitted against time-course gene expression for control and treated samples [1]. The differential expression of a gene has been discovered by applying empirical Bayes moderated F-statistics on the spline regression model's coefficient differences.

The mathematical natural cubic spline regression model is defined as:

$$f = f(t, z) = s_0 + \sum_{i=1}^{n} s_i B_i(t - t_0) + z(d_0 + \sum_{i=1}^{n} d_i B_i(t - t_0)) \tag{1}$$

where s_i and d_i is the reference group's spline coefficient and compared group's differential spline coefficient, respectively and $B_i(t - t_0)$ is spline base function $\forall_i = 1, 2, \ldots, n$ with t_0 being the first measurement time.

$$f = \begin{cases} f_{\text{reference,}} & \text{if } z = 0 \\ f_{\text{compared,}} & \text{if } z = 1 \end{cases} \tag{2}$$

The detection of DE genes using splineTimeR has been carried out by setting the Benjamini-Hochberg [2] adjusted p-value threshold to 0.05 and with a degree of freedom of 4 for all genes. Top regulated genes have been identified as differentially expressed and used for our subsequent analysis in module discovery.

Additionally, to identify the DE genes at each time point, we have employed the limma package [3]. Limma utilizes empirical Bayes smoothing on the estimated fold-changes and standard errors from a linear model fitting to assesses the differential gene expression across a pair conditions.

A linear model can be defined as:

$$\beta[e_g] = D \cdot \Gamma_g, \tag{3}$$

where e_g represents a vector of expression values and D represents the design matrix which maps these vector to some coefficient of interest Γ_g for each gene g. A contrast matrix was also used for computing log_2-fold-changes and t-statistics by allowing all possible pairwise comparisons between gene samples. Furthermore, test-statistics are obtained to perform gene ranking. Normalized expression values for 3 replicates at each of the 5-time points were used for differential expression analysis.

2 Dirichlet Process Gaussian Process mixture model (DPGP)

We have utilized a non-parametric model-based approach, the Dirichlet Process Gaussian Process mixture model (DPGP), presented by McDowell *et al.* to obtain gene modules from the univariate time-course expression data [4]. It can simultaneously model cluster number with a Dirichlet process (DP) and temporal dependencies with Gaussian Processes (GP). DPGP uses a Bayesian non-parametric model for time course paths $P \in R^{N \times T}$, where N is the number of genes and T is the number of time points (see the supplement for a detailed description of DPGP method).

A generative Dirichlet process (DP) mixture model is defined as

$$V_i \sim DP(\delta, G) \tag{4}$$

Here, DP represents a draw which generates latent variables V_i for a cluster i with δ being the concentration parameter and G being the base distribution, and the observation distribution $M_j \sim p(. \mid V_i)$ is specified with a Gaussian Process (GP) for gene $j \in \{1, 2, ..., N\}$. Cluster-specific parameter values V_i is obtained from conditional probability $p(V_i \mid V_{\neg i})$ on all other variables except V_i by integrating draws from DP. The within-cluster parameters of the trajectories for a cluster i, i.e. V_i=$\{\mu_i, \ell_i, \zeta_i, \sigma_i^2\}$ is described as $\mu_i \sim GP(\mu_0, L)$ with $\ell_i, \zeta_i \sim \ln \mathcal{N}(0, 1)$ and σ_i^2 being an inverse gamma function of shape and rate of the trajectory.

DPGP uses a cluster-specific positive definite Gram matrix L quantifying similarity between each and every time point $T_x, T_{x'}$ for a cluster i. This matrix is computed using a squared exponential (SE) function, an isotropic Mercer kernel, defined as:

$$L_i(T_x, T_{x'}) = \zeta_i^2 \exp\left\{-\frac{\|T_x - T_{x'}\|_2}{2\ell_i^2}\right\} \tag{5}$$

The hyperparameter ℓ_i, ζ_i corresponds to input space distance among data points having correlated output (*characteristic length scale*), gene expression path variance over time (*signal variance*), respectively. Thus, the probability distribution of each observation M_j, corresponding to a specific cluster GP including unique marginal variance, is characterized by

$$M_j \sim \mathcal{N}(\mu_i, L_i + \sigma_i^2 I) \tag{6}$$

Choice of kernel functions always impact the quality of the result. Therefore, we have also applied *standard periodic* (stdp), *Matérn52* kernel functions in order to find the optimal kernel for our dataset. The stdp kernel can be defined as a function of input points which allows us to model repeating kernel functions [5]. A stdp kernel with length scale ℓ to limit extrapolation only upto ℓ units away from data and a vector of periods T_i is represented as:

$$k(x, y) = \sigma^2 \exp\left[-\frac{1}{2}\sum_{i=1}^{input\_dim}\left(\frac{\sin(\frac{\pi}{T_i}(x_i - y_i))}{\ell_i}\right)^2\right] \tag{7}$$

The collection of Matérn kernels is a generalized version of the Radial Basis Functions (RBFs). The function is a Mercer kernel which contains additional positive parameters (ℓ), and ν that determines the smoothness of the output curve [6]. The Matérn covariance between two vectors separated by d distance units is given by:

$$k(x_i, x_j) = \frac{1}{\Gamma(\nu)2^{\nu-1}} \left(\frac{\sqrt{2\nu}}{\ell} d(x_i, x_j) \right)^{\nu} K_{\nu} \left(\frac{\sqrt{2\nu}}{\ell} d(x_i, x_j) \right), \tag{8}$$

where $d(.,.)$ is the Euclidean distance, $K_{\nu}(.)$ is a modified Bessel function and $\Gamma(.)$ is the gamma function. We chose a particular value of $\nu = 5/2$ and a unit length scale (ℓ), thus making the function twice differentiable and resulting in the final Matérn52 kernel as:

$$k(r) = \sigma^2 (1 + \sqrt{5}r + \frac{5}{3}r^2) \exp(-\sqrt{5}r), \text{where } r = \sqrt{\sum_{i=1}^{input\_dim} \frac{(x_i - y_i)^2}{\ell_i^2}} \tag{9}$$

References

[1] Michna, A. *et al.* Natural cubic spline regression modeling followed by dynamic network reconstruction for the identification of radiation-sensitivity gene association networks from time-course transcriptome data. *PloS one* **11** (2016).

[2] Thissen, D., Steinberg, L. & Kuang, D. Quick and easy implementation of the benjamini-hochberg procedure for controlling the false positive rate in multiple comparisons. *Journal of educational and behavioral statistics* **27**, 77–83 (2002).

[3] Ritchie, M. E. *et al.* limma powers differential expression analyses for RNA-sequencing and microarray studies. *Nucleic Acids Research* **43**, e47–e47, DOI: 10.1093/nar/gkv007 (2015).

[4] McDowell, I. C. *et al.* Clustering gene expression time series data using an infinite gaussian process mixture model. *PLoS computational biology* **14**, e1005896 (2018).

[5] Schölkopf, B., Smola, A. J., Bach, F. *et al.* *Learning with kernels: support vector machines, regularization, optimization, and beyond* (MIT press, 2002).

[6] Rasmussen, C. E. Gaussian processes in machine learning. In *Summer school on machine learning*, 63–71 (Springer, 2003).

[7] Gu, Z., Eils, R. & Schlesner, M. Complex heatmaps reveal patterns and correlations in multidimensional genomic data. *Bioinformatics* **32**, 2847–2849, DOI: 10.1093/bioinformatics/btw313 (2016).

[8] Wickham, H. *ggplot2: Elegant Graphics for Data Analysis* (Springer-Verlag New York, 2016).

[9] Dusa, A. *venn: Draw Venn Diagrams* (2020). R package version 1.9.

[10] Shannon, P. *et al.* Cytoscape: a software environment for integrated models of biomolecular interaction networks. *Genome research* **13**, 2498–2504 (2003).

Additional information

We have utilized the following software packages in our present study: Inkscape (version 1.0.2), ComplexHeatmap [7], ggplot2 [8], venn [9], Cytoscape [10] (version 3.8.2) to produce the images.

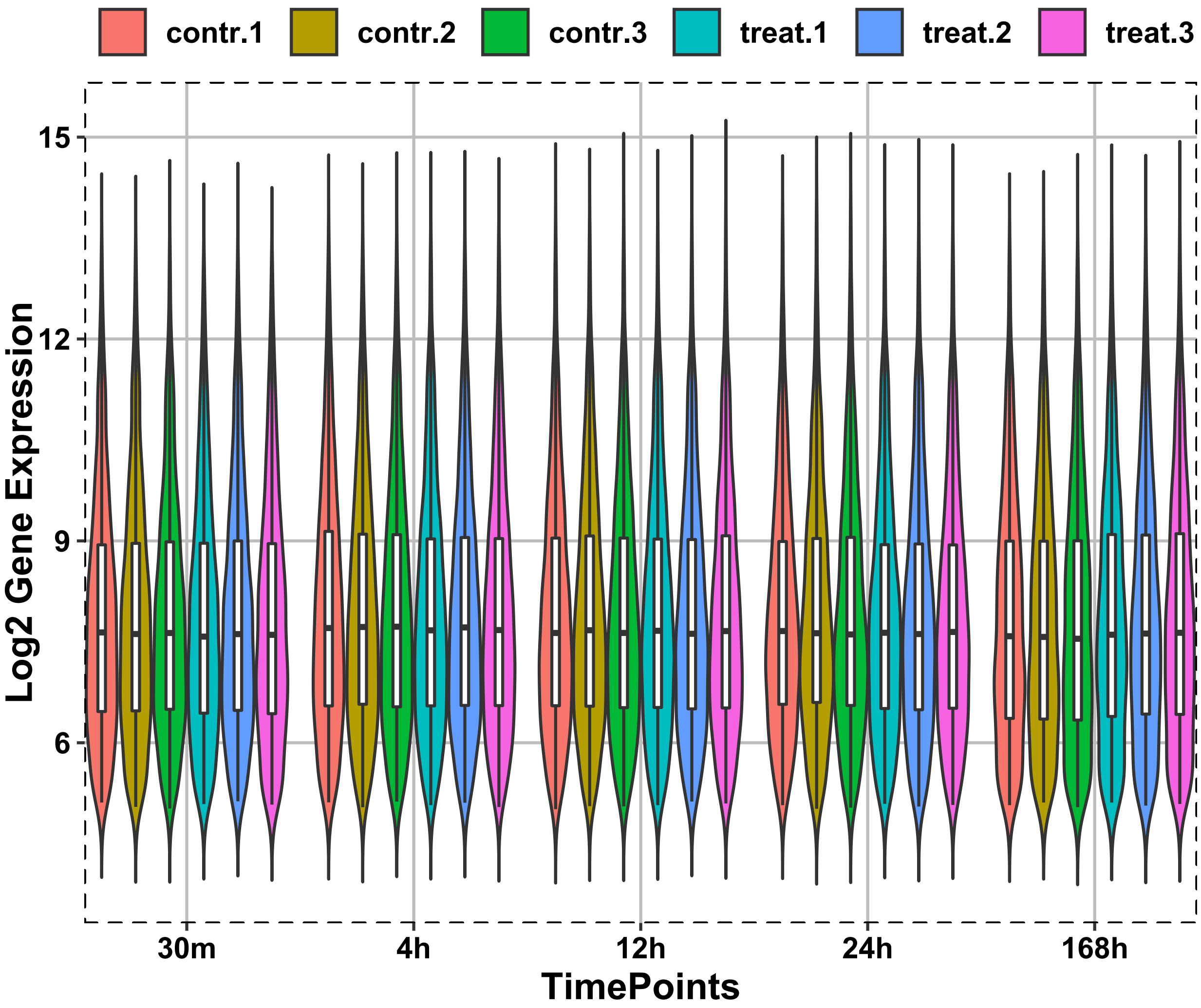

Figure 1: The figure shows the box and violin plots of cubic spline normalized gene expression of the differentially expressed genes.

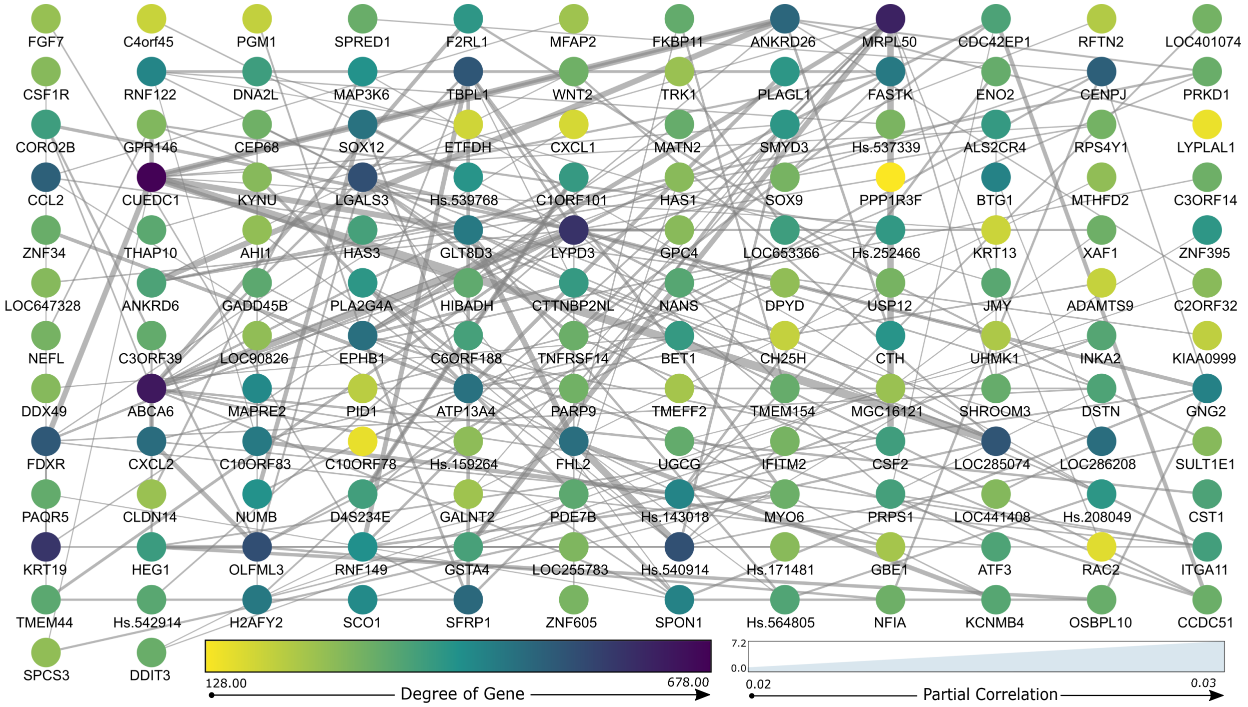

Figure 2: The figure shows the reconstructed Gene Association Network using splineNetRecon with top 150 edges based on partial correlation score with degree centrality measure. The figure has been produced through the Cytoscape software (version 3.8.2) [10].

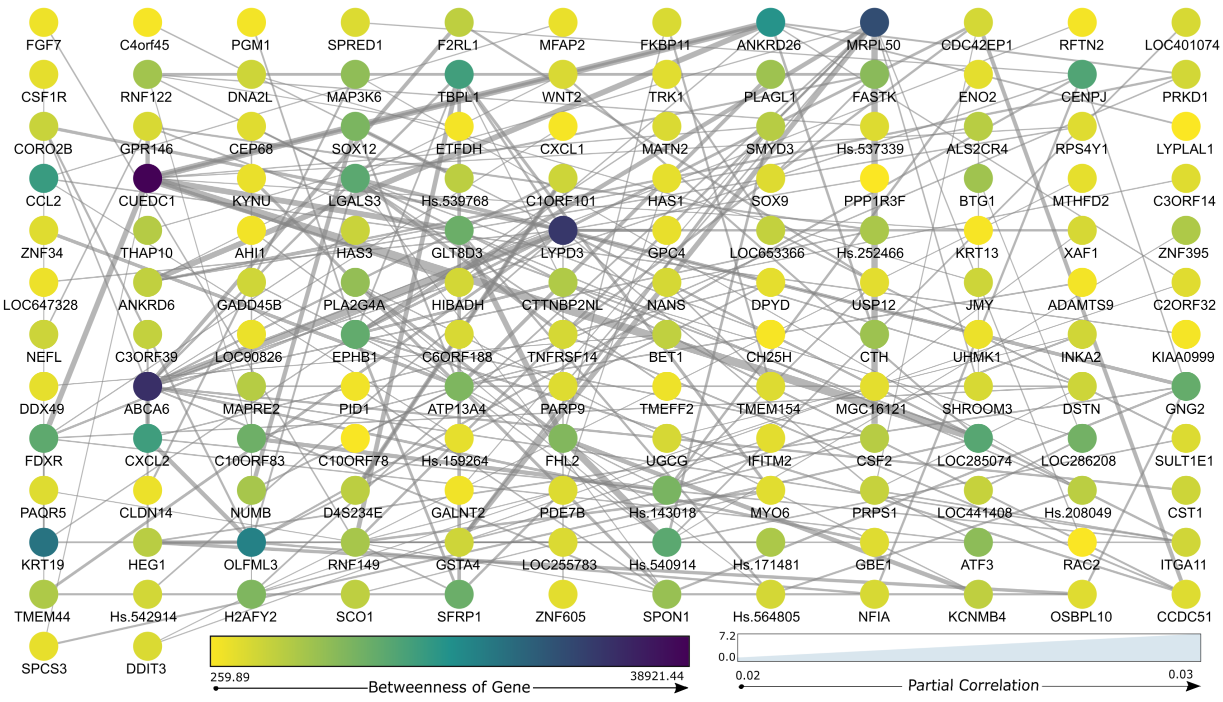

Figure 3: The figure shows the reconstructed Gene Association Network using splineNetRecon with top 150 edges based on partial correlation score with betweenness centrality measure. The figure has been produced through the Cytoscape software (version 3.8.2) [10].

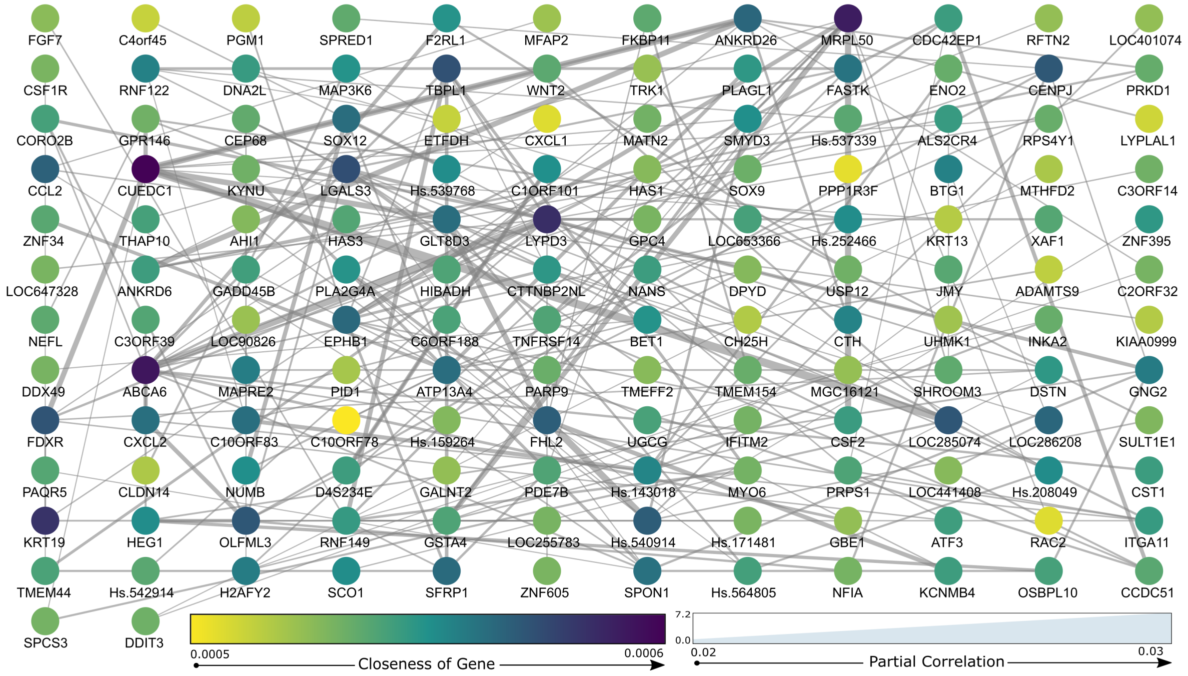

Figure 4: The figure shows the reconstructed Gene Association Network using splineNetRecon with top 150 edges based on partial correlation score with closeness centrality measure. The figure has been produced through the Cytoscape software (version 3.8.2) [10].

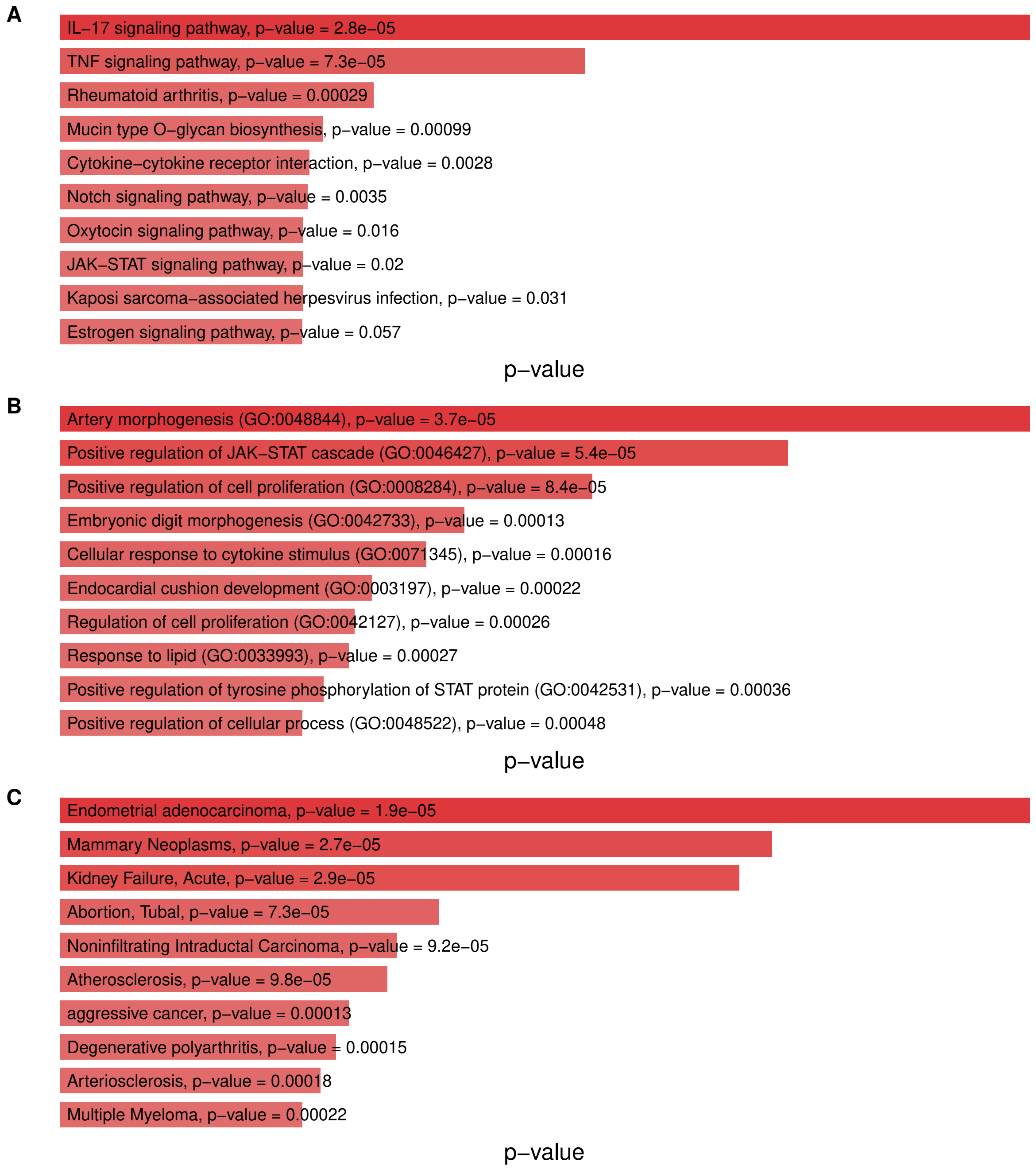

Figure 5: The figure shows the KEGG Pathway (A), Biological Processes (B), and DisGeNET analysis (C) of the key module 2.

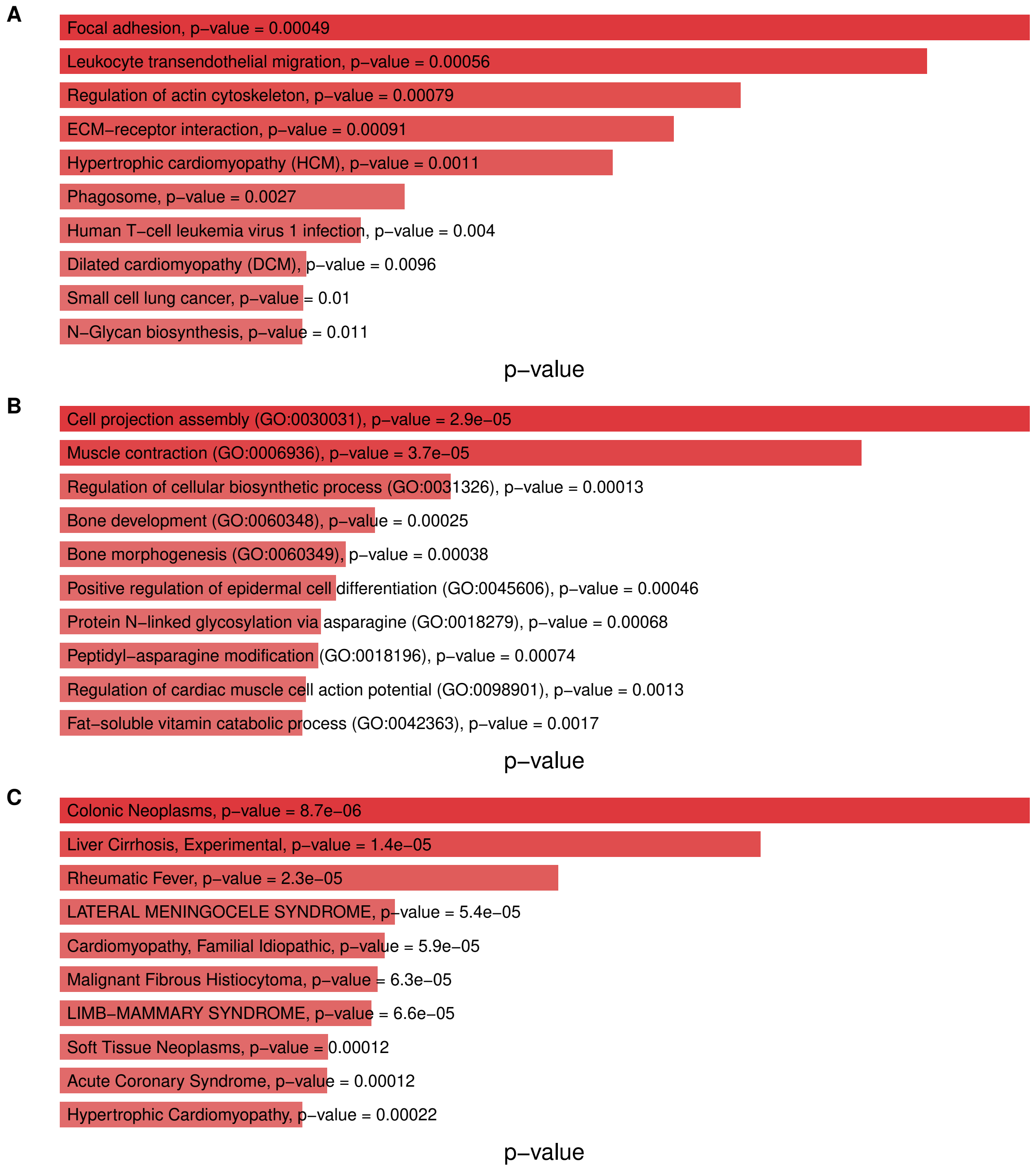

Figure 6: The figure shows the KEGG Pathway (A), Biological Processes (B), and DisGeNET analysis (C) of the key module 3.

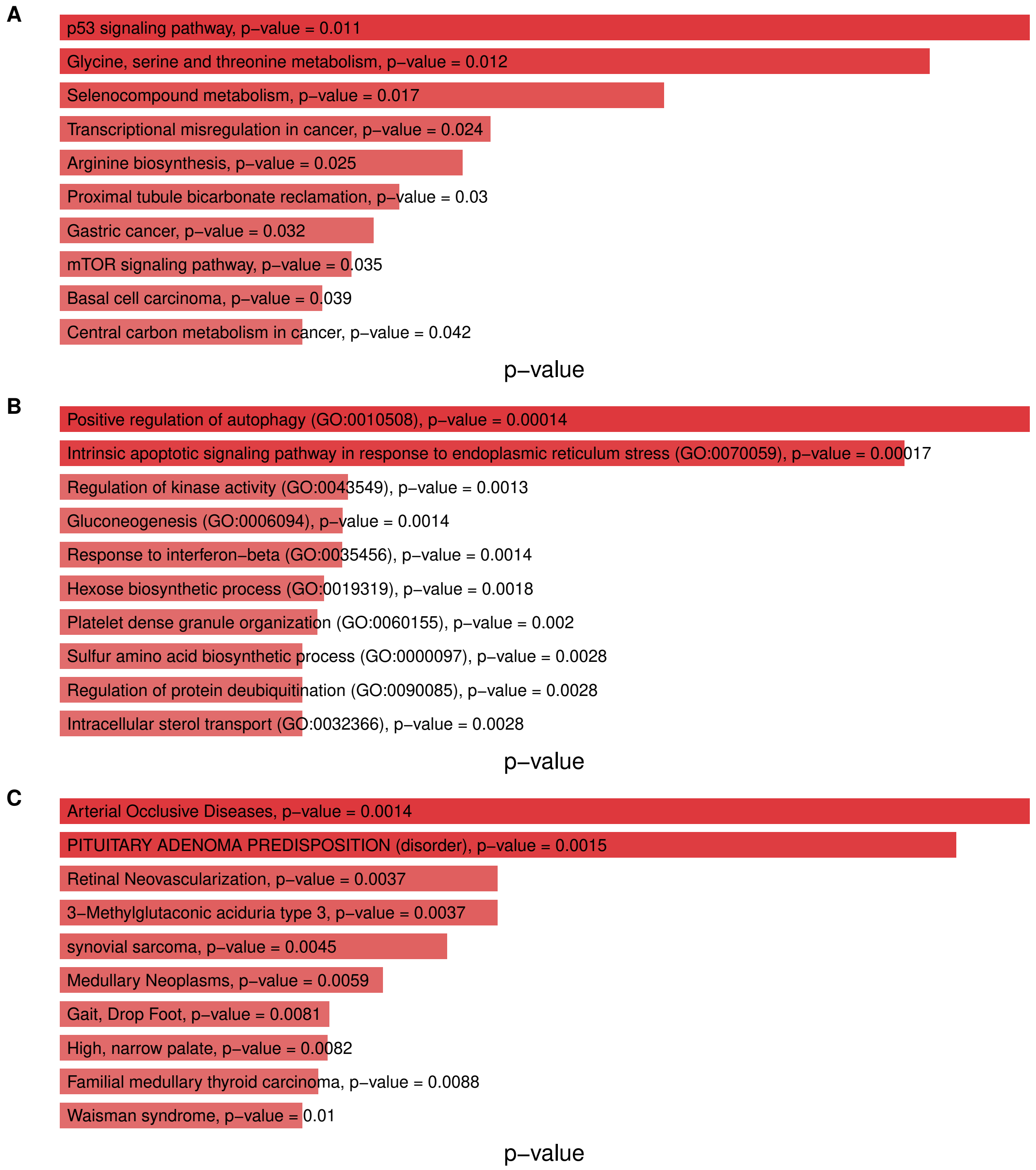

Figure 7: The figure shows the KEGG Pathway (A), Biological Processes (B), and DisGeNET analysis (C) of the key module 4.

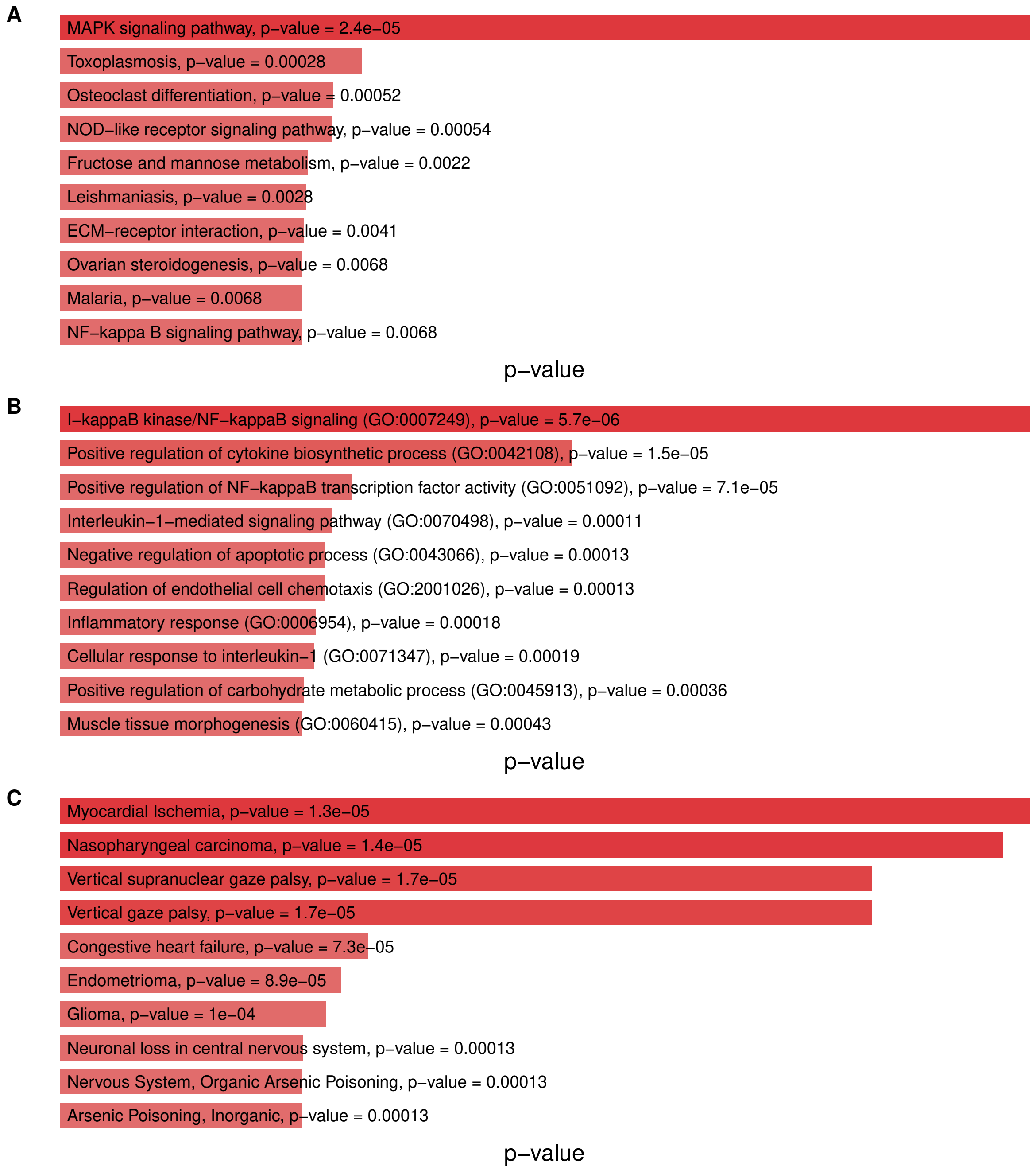

Figure 8: The figure shows the KEGG Pathway (A), Biological Processes (B), and DisGeNET analysis (C) of the key module 5.

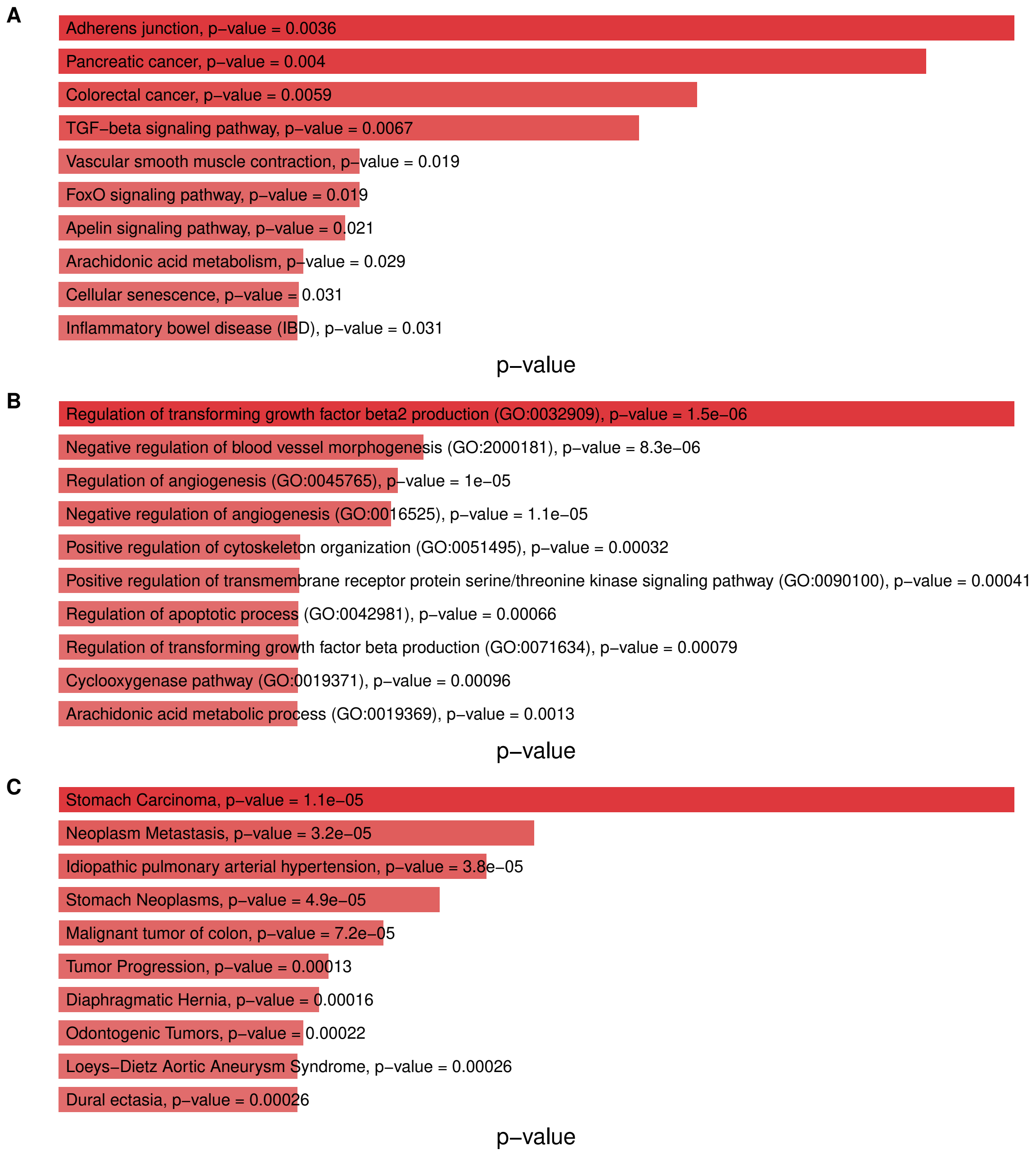

Figure 9: The figure shows the KEGG Pathway (A), Biological Processes (B), and DisGeNET analysis (C) of the key module 6.

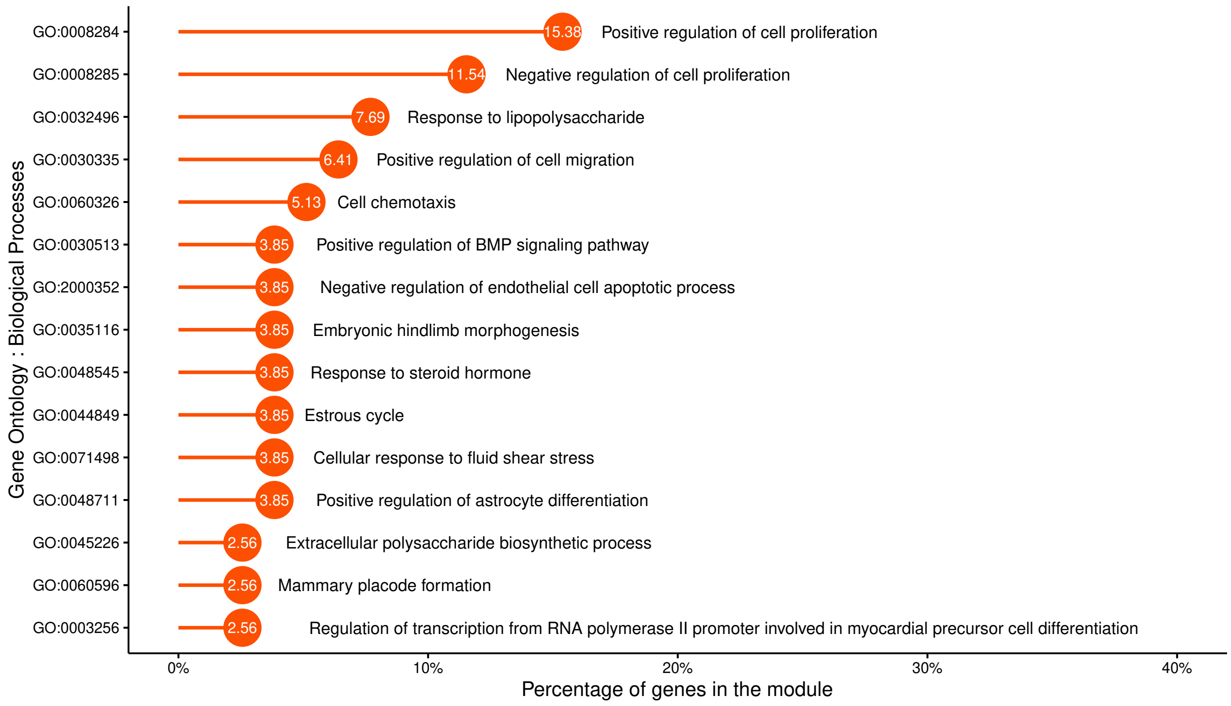

Figure 10: The figure shows the lollipop plot describing the percentage of genes in key module 2, contributing to the top 15 gene ontology terms.

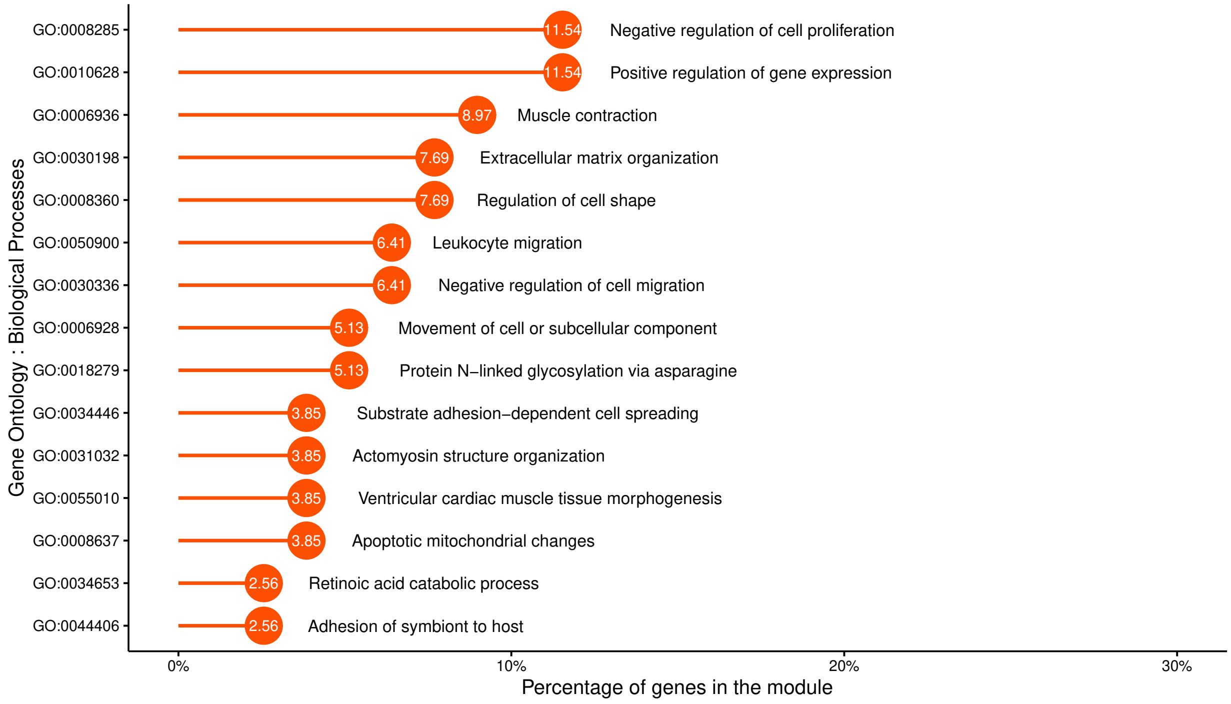

Figure 11: The figure shows the lollipop plot describing the percentage of genes in key module 3, contributing to the top 15 gene ontology terms.

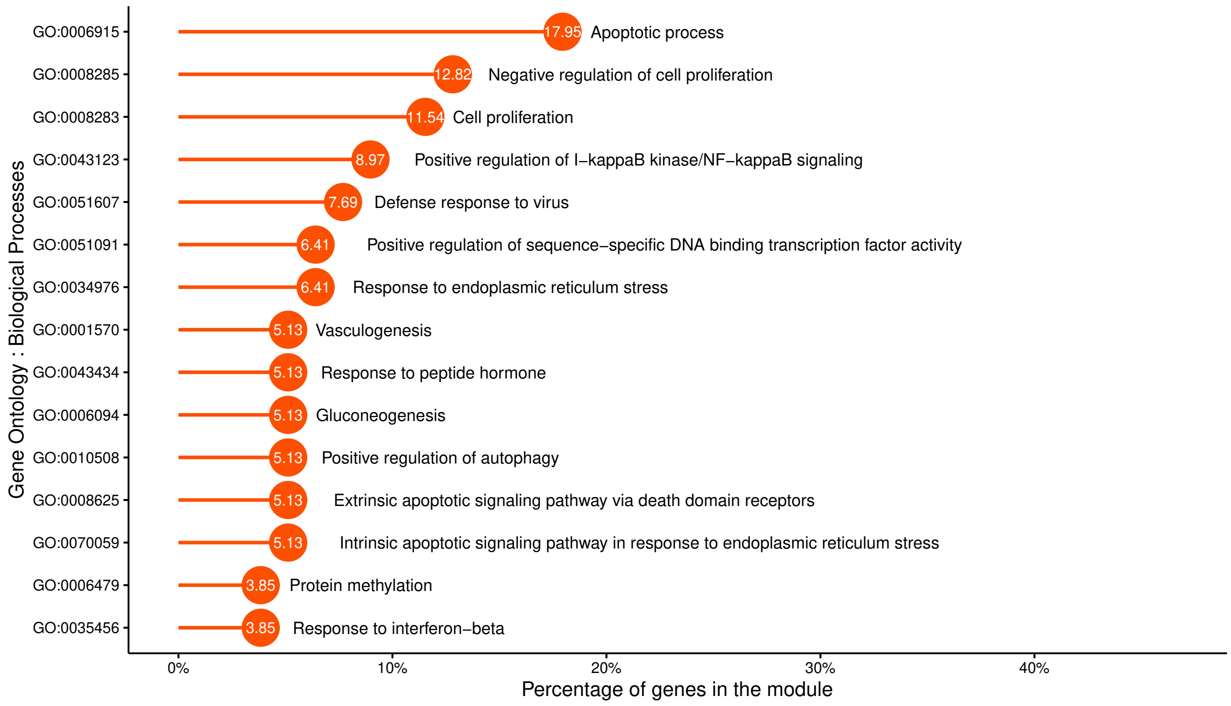

Figure 12: The figure shows the lollipop plot describing the percentage of genes in key module 4, contributing to the top 15 gene ontology terms.

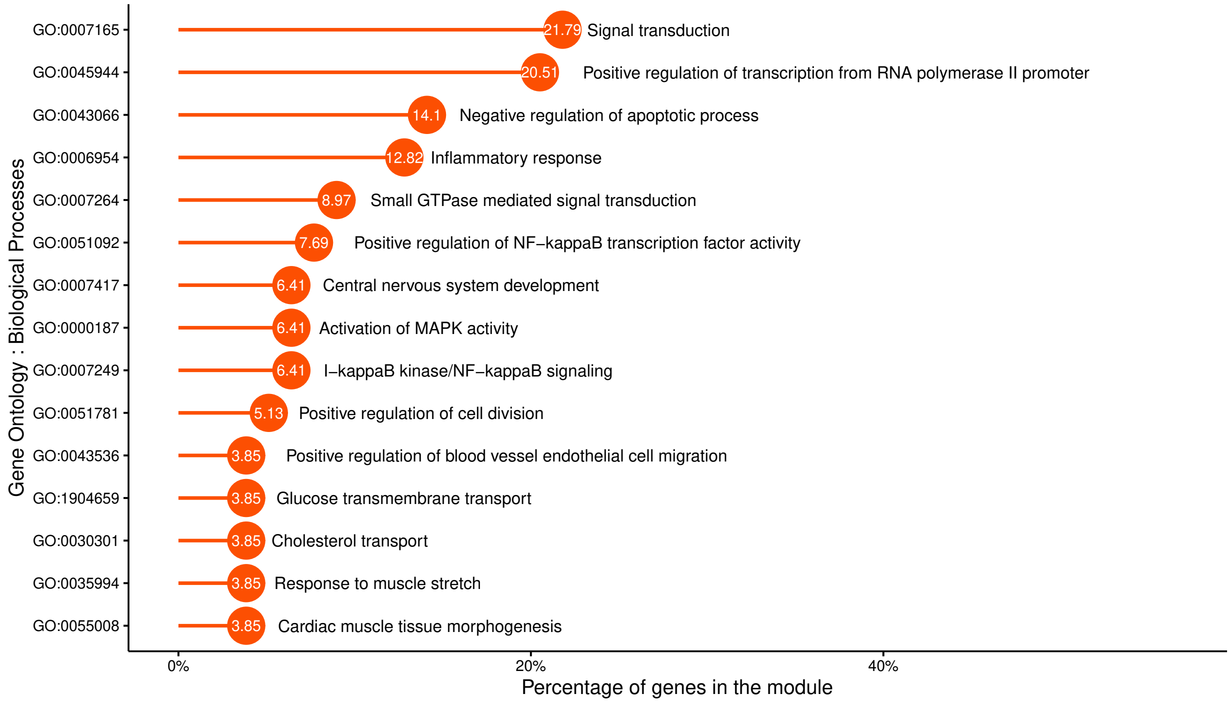

Figure 13: The figure shows the lollipop plot describing the percentage of genes in key module 5, contributing to the top 15 gene ontology terms.

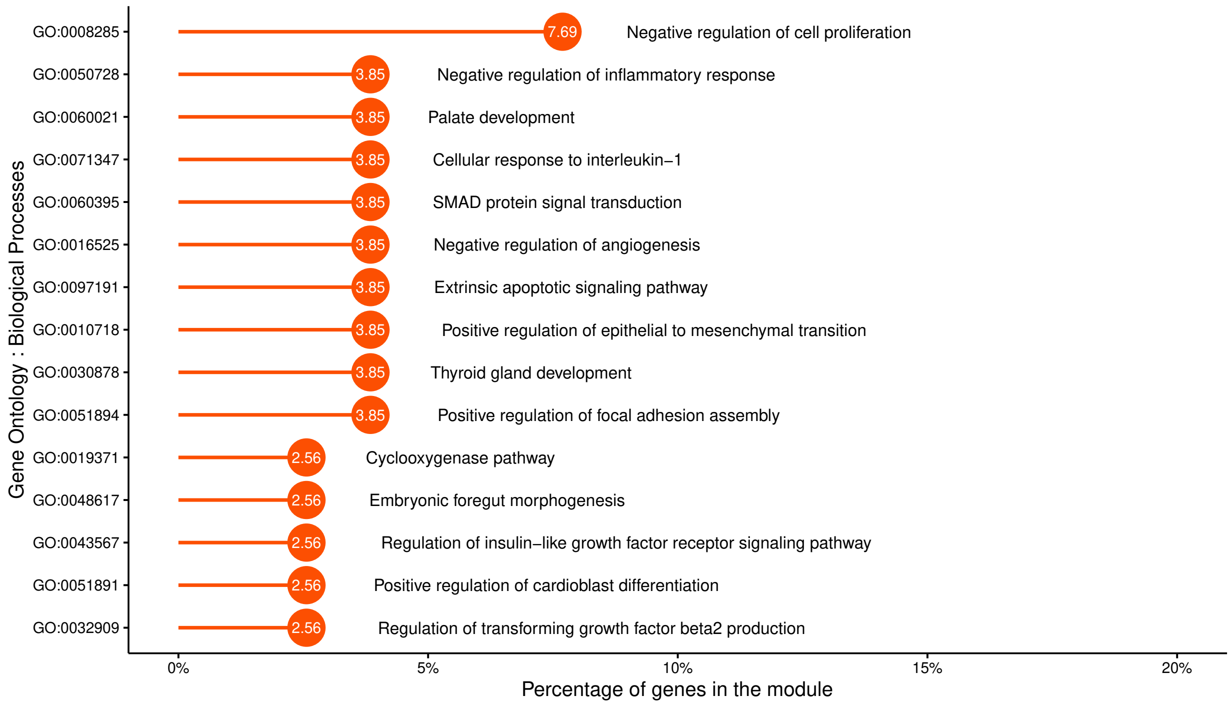

Figure 14: The figure shows the lollipop plot describing the percentage of genes in key module 6, contributing to the top 15 gene ontology terms.

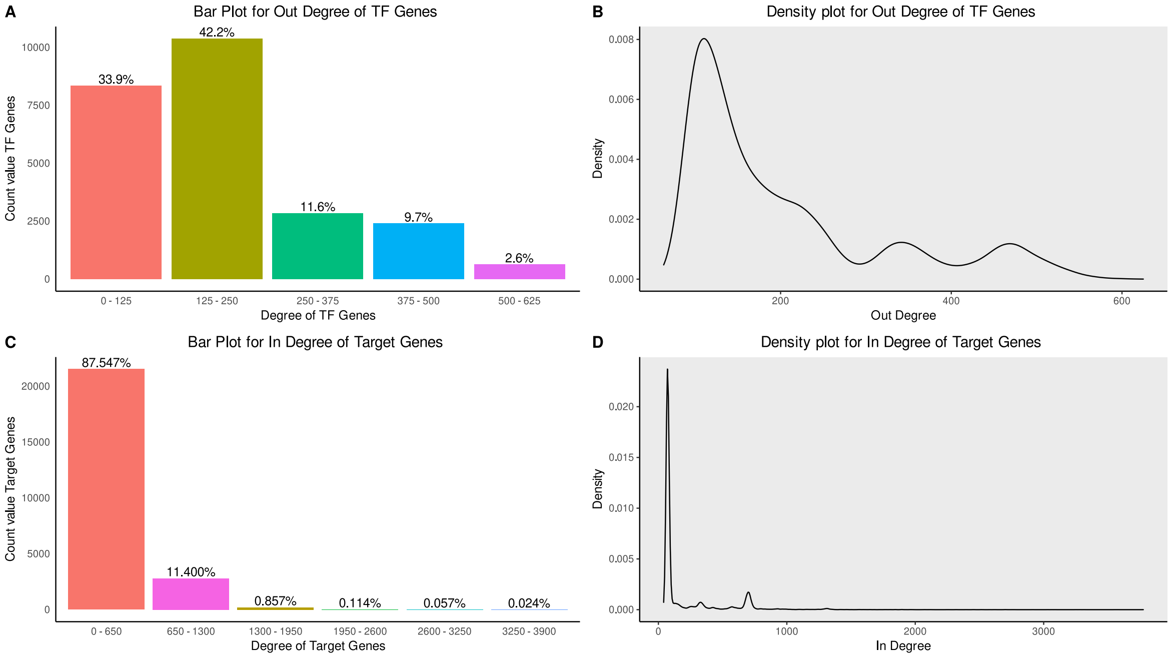

Figure 15: The figure shows the in- and out-degree distribution of the target genes and the transcription factors.

Table 1: Identified regulators for the 21 target DE genes with highly significant interaction score within the whole GRN obtained from dynGENIE3.

**TF Gene**	**Target Gene**	**dynGENIE3 Score**
CYB5A	B3GALNT1	1
PCBP3	PFTK1	1
LOC285307	FST	1
LOC646400	SHC3	1
PAX6	LOC645253	1
LOC645789	LOC92312	1
ALK	TRPC4	1
CYP4Z1	PTS	1
ETV5	ARHGAP18	1
TNFRSF10D	CLDN11	0.55895705
HLA-DRB1	ALPL	0.535035266
ADAMTS10	PID1	0.527034107
TMEM16F	CNN1	0.518205817
PTDSS1	GOT1	0.509718007
PXN	FN1	0.5051634
CSNK2B	HOXA2	0.505054021
ENTPD8	DDX41	0.500027846
LOC650678	DDX41	0.499972154
PTPRQ	HOXA2	0.494945979
LOC729744	FN1	0.4948366
MKKS	GOT1	0.490281993
BBS10	CNN1	0.481794183
DEPDC2	PID1	0.472965893
PSMD5	ALPL	0.464964734
NME3	CLDN11	0.44104295
TSGA13	PLXNA3	0.353027304
LOC642568	PLEKHA6	0.351180179
BDNF	RBPJ	0.345587425
ZNF77	SRD5A1	0.344367844
KRIT1	RBPJ	0.339378292
XAB1	PLXNA3	0.332847396
LOC648976	SRD5A1	0.330290016
TUBB4Q	PLEKHA6	0.328000988
76P	SRD5A1	0.32534214
LOC648981	PLEKHA6	0.320818834
GALR3	RBPJ	0.315034283
TPD52L2	PLXNA3	0.3141253

Table 2: List of potential transcription factor reported by our study. The common transcription factors obtained from REGGAE and two independent studies are reported with bold font.

**Potential**	**TFs**
**SREBF1**	RING1
**SMAD3**	TAF15
**EGR2**	DNMT1
**HOXC13**	ELL2
**ZNF281**	DACH1
**NKX3-1**	MSX1
**NR4A2**	NCAPG
**NFKB1**	PBX3
**E2F4**	PEX2
**SOX9**	TOP2B
**NFYB**	HOXA2
**RFX5**	LIN54
**ID3**	JARID2
**LYL1**	HES4
**HOXA5**	HEXIM1
**FOXF1**	TRIM22
**ID1**	NOTCH1
**RREB1**	LMNA
**KLF6**	ZNF280D
**MYB**	PRPF4
**TBX3**	ZNF34
**IRF1**	ZNF792
**STAT1**	
**ARNT2**	
**RELB**	
**ZBTB20**	
**RBPJ**	
**E2F7**	
**FOSL1**	
**HOXA4**	
**FOXO1**	
**ATF3**	
**RARB**	
**SMAD6**	
**ZNF395**	
**CREB3L2**	
**TCF3**	
**HIVEP1**	
**POU2F2**	
**NR1H4**	
**FOSB**	
**EGR1**	
**KLF13**	

Reported Silhouette width for selecting the best parameters in DPGP

Table 3 (A)

Squared Exponential Kernel					
α = 1.0, MAP clustering, L-BFGS optimization technique					
	βIG = 1	βIG = 2	βIG = 3	βIG = 4	βIG = 5
αIG = 1	0.4497	0.5448	0.5758	0.5697	0.5647
αIG = 2	0.4299	0.5755	0.5212	0.5118	0.5655
αIG = 3	0.5530	0.5520	0.5910	0.5963	0.5906
αIG = 4	0.6185	0.5965	0.5711	0.5146	0.5292
αIG = 5	0.5195	0.5179	0.5458	0.5660	0.5458
αIG = 6	0.5893	0.5268	0.5895	0.4978	0.5400
αIG = 7	0.4868	0.5844	0.6011	0.5853	0.5877
αIG = 8	0.5662	0.6039	0.5350	0.5531	0.5311
αIG = 9	0.5454	0.5620	0.5986	0.5957	0.5607
αIG = 10	0.4994	0.5506	0.6142	0.5838	0.5233
αIG = 11	0.5248	0.5400	0.5450	0.5708	0.6023
αIG = 12	0.4882	0.5734	0.4783	0.5122	0.5248
αIG = 13	0.5969	0.6108	0.5310	0.5395	0.6047
αIG = 14	0.5541	0.5759	0.5415	0.5486	0.6019
αIG = 15	0.4538	0.5079	0.6112	0.6091	0.5152

Table 3 (B)

Matern52 Kernel					
α = 1.0, MAP clustering, L-BFGS optimization technique					
	βIG = 1	βIG = 2	βIG = 3	βIG = 4	βIG = 5
αIG = 1	0.505	0.5375	0.5945	0.5745	0.5942
αIG = 2	0.5999	0.485	0.5985	0.4847	0.5932
αIG = 3	0.5996	0.5215	0.5616	0.5519	0.5843
αIG = 4	0.5428	0.5466	0.5448	0.5889	0.564
αIG = 5	0.5207	0.6125	0.5937	0.5956	0.5874
αIG = 6	0.5035	0.5533	0.5626	0.5001	0.5705
αIG = 7	0.545	0.4416	0.5681	0.5487	0.5754
αIG = 8	0.5708	0.5782	0.5737	0.543	0.5099
αIG = 9	0.5832	0.5534	0.5613	0.5652	0.5523
αIG = 10	0.6102	0.4829	0.5082	0.5689	0.4738
αIG = 11	0.5138	0.4591	0.4927	0.5346	0.4531
αIG = 12	0.5488	0.6045	0.5317	0.4713	0.5397
αIG = 13	0.5117	0.5605	0.5553	0.5364	0.5567
αIG = 14	0.4729	0.4859	0.5338	0.5246	0.5219
αIG = 15	0.6117	0.6	0.5199	0.5008	0.5258

Table 3 (D)

Optimization Technique	
α = 1.0, αIG = 4, βIG = 1, MAP clustering	
FMIN_TNC	0.4954
L-BFGS	0.6185
SCG	0.5738

Table 3 (E)

Clustering Technique	
α = 1.0, αIG = 4, βIG = 1, L-BFGS optimization technique	
Least Square	0.5988
MPEAR	0.4377
MAP	0.6185

Table 3 (F)

Tuning concentration parameter (α)	
αIG = 4, βIG = 1, MAP clustering, L-BFGS optimization	
α = 0.1	0.5902
α = 0.2	0.6046
α = 0.3	0.3851
α = 0.4	0.5407
α = 0.5	0.4986
α = 0.6	0.4415
α = 0.7	0.6077
α = 0.8	0.6122
α = 0.9	0.3604
α = 1.0	0.6185
α = 1.1	0.5350
α = 1.2	0.5696
α = 1.3	0.6169
α = 1.4	0.4977
α = 1.5	0.5835
α = 1.6	0.5532
α = 1.7	0.4584
α = 1.8	0.5361
α = 1.9	0.4910
α = 2.0	0.5152

Reported Silhouette width for selecting the best parameters in DPGP Contd…

Table 3 (C)

Standard Periodic Kernel					
α = 1.0, MAP clustering, L-BFGS optimization technique					
	βIG = 1	βIG = 2	βIG = 3	βIG = 4	βIG = 5
αIG = 1	0.3647	0.4629	0.3859	0.3946	0.4622
αIG = 2	0.4234	0.4525	0.4504	0.4586	0.4789
αIG = 3	0.3893	0.3475	0.4545	0.5516	0.4709
αIG = 4	0.3696	0.4498	0.3391	0.5317	0.5174
αIG = 5	0.5032	0.5166	0.478	0.3663	0.4183
αIG = 6	0.4576	0.418	0.4621	0.3779	0.423
αIG = 7	0.4081	0.5059	0.4202	0.4081	0.452
αIG = 8	0.3883	0.4633	0.4308	0.4098	0.4419
αIG = 9	0.4876	0.3877	0.4987	0.3843	0.3311
αIG = 10	0.4563	0.4432	0.371	0.4236	0.3392
αIG = 11	0.4689	0.4111	0.3852	0.3475	0.3303
αIG = 12	0.4173	0.4703	0.2867	0.3924	0.3093
αIG = 13	0.425	0.4826	0.3743	0.2492	0.2989
αIG = 14	0.4178	0.355	0.4058	0.2811	0.3244
αIG = 15	0.4459	0.3917	0.389	0.3305	0.363

Table 3 (G)

Other Clustering Techniques	
(with 10 clusters)	
Hierarchical Clustering, complete	0.2937
Hierarchical Clustering, average	0.3598
K-Means Clustering	0.3189
Tsclust [1], complete	0.2965
Tsclust [1], average	0.3426
BHC [2]	0.2062
Mclust [3]	-0.1048
TmixClust [4]	0.2200
GMM, EM [5]	0.2589
GIMM [6]	0.2761

Clustering Technique
MAP = Maximum a posteriori
MPEAR = Posterior expected adjusted Rand
least_squares = Minimize squared distance between clustering and posterior similarity matrix

Optimization Technique
FMIN_TNC = Truncated Newton algorithm
SCG = Stochastic Conjugate Gradient
L-BFGS = Limited-Memory Broyden-Fletcher-Goldfarb-Shanno

References

[1] Montero P, Vilar JA (2014). TSclust: An R Package for Time Series Clustering.
[2] Savage R, Cooke E, Darkins R, Xu Y (2020). BHC: Bayesian Hierarchical Clustering.
[3] Scrucca L, et al. “mclust 5: clustering, classification and density estimation using Gaussian finite mixture models.
[4] Golumbeanu M (2020). TMixClust: Time Series Clustering of Gene Expression with Gaussian Mixed-Effects Models and Smoothing Splines.
[5] Lampros Mouselimis (2020). ClusterR: Gaussian Mixture Models, K-Means, Mini-Batch-Kmeans, K-Medoids and Affinity Propagation Clustering.
[6] Medvedovic M, Yeung KY, Bumgarner RE. Bayesian mixture model based clustering of replicated microarray data. Bioinformatics.

Table 4: interactions among the regulators and the target genes of the inferred GRN with top scores.

	TF Gene	Target Gene	Score
1	SPRR3	DHX38	1.000
2	SPRR3	NR4A1	1.000
3	OSBP2	CEP57	1.000
4	TMEM55A	DHRSX	1.000
5	TRAF2	LOC644325	1.000
6	CXCR6	LOC648044	1.000
7	CCDC51	MRPS17	1.000
8	EHD4	PSIP1	1.000
9	NODAL	LOC390637	1.000
10	C5orf37	TOP3A	1.000
11	PLXNA4B	IGFBPL1	1.000
12	KCTD3	TCL6	1.000
13	OPTC	MSH3	1.000
14	LOC645576	GAS2L3	1.000
15	RDH10	LOC643836	1.000
16	LOC493869	RDH14	1.000
17	CIB1	AGTRL1	1.000
18	POLA2	FAM112B	1.000
19	LOC645262	PELO	1.000
20	RND2	SLITRK6	1.000
21	FGFR1OP2	LOC91661	1.000
22	LOC402110	TTLL3	1.000
23	METRN	TRIAP1	1.000
24	LOC645681	GALK2	1.000
25	ATP10B	ZNF670	1.000
26	EAF1	ADH4	1.000
27	FLJ40712	AYP1p1	1.000
28	CXCR7	PARS2	1.000
29	LOC441052	C16orf69	1.000
30	ANKRD1	OR4C6	1.000
31	UNQ6975	LOC652697	1.000
32	DMRTB1	CCDC85B	1.000
33	DMRTB1	XRCC6BP1	1.000
34	TMCO3	LOC650850	1.000
35	MYO1B	GALNTL1	1.000
36	LOC645451	C21orf57	1.000
37	PRSSL1	PAFAH1B2	1.000
38	CCDC124	HIST1H2BG	1.000
39	POLR3C	LMO7	1.000
40	NFKBIA	NTN4	1.000
41	PSMD13	LOC201181	1.000
42	LOC647843	C5orf38	1.000
43	CYB5A	B3GALNT1	1.000
44	DUSP21	C14orf162	1.000
45	ANKRD31	C20orf86	1.000
46	RUFY1	SRRM2	1.000
47	TMPRSS12	ETNK2	1.000
48	CLEC14A	DDX19B	1.000
49	LOC648603	SYT8	1.000
50	TRAPPC6B	CD4	1.000
51	LOC653554	LOC641875	1.000
52	RUSC2	URP2	1.000
53	CCDC115	JARID1A	1.000
54	IVD	C1orf35	1.000
55	C7orf13	DEPDC1B	1.000
56	MRPL10	LOC374395	1.000
57	L1CAM	MED8	1.000
58	YTHDF3	LOC127406	1.000
59	PRLH	FLJ36070	1.000
60	LOC643809	APIP	1.000
61	LOC644325	LOC653577	1.000
62	DLG3	ZNF774	1.000
63	AVIL	MAP3K2	1.000
64	FLJ10781	LOC645212	1.000
65	DDHD1	LOC220115	1.000
66	HS6ST2	LOC641815	1.000
67	PQLC2	LOC642093	1.000
68	OR52N1	TAOK1	1.000
69	LOC653342	LOC643334	1.000
70	FLJ42418	LOC647942	1.000
71	LOC646447	HIST1H3A	1.000
72	EIF3S6IP	DLD	1.000
73	ENDOGL1	POLR3H	1.000
74	ELAVL4	SLC16A5	1.000
75	SNORD54	SCAND1	1.000
76	PCBP3	PFTK1	1.000
77	NET1	MCTP2	1.000
78	TMEM61	LOC644808	1.000
79	RGS9BP	LOC650235	1.000
80	LOC652216	ERBB2IP	1.000
81	FLJ31438	MTX1	1.000
82	CD58	PAK3	1.000
83	LOC442582	LOC642130	1.000
84	LOC650632	MAEL	1.000
85	DDHD2	CDK3	1.000
86	LOC285307	FST	1.000

Table 4: interactions among the regulators and the target genes of the inferred GRN with top scores. (Contd..)

	TF Gene	Target Gene	Score
87	SMCR7L	LOC644884	1.000
88	LOC644403	LOC641693	1.000
89	SLC17A2	PMS2L2	1.000
90	LOC441098	TSPAN31	1.000
91	KIAA2026	LOC652659	1.000
92	RHCE	LOC165186	1.000
93	MUC17	C18orf17	1.000
94	LOC389669	KIAA0226	1.000
95	FLJ31875	PCDHB10	1.000
96	LOC650843	C9orf31	1.000
97	OR5AU1	PTGDS	1.000
98	LOC647058	NDUFS2	1.000
99	OR10S1	LOC652479	1.000
100	C17orf39	LOC652127	1.000
101	RPL10L	LOC642646	1.000
102	ATOH1	OVOL2	1.000
103	LOC649317	LOC285900	1.000
104	SAMD12	LOC339229	1.000
105	RPL37	LOC653751	1.000
106	LOC93349	SNW1	1.000
107	LOC643378	SLC5A7	1.000
108	LOC647407	SYT9	1.000
109	LOC345643	KIAA0495	1.000
110	PIN4	SUFU	1.000
111	LOC646400	SHC3	1.000
112	ZNF256	ZNF233	1.000
113	CCDC126	RASL12	1.000
114	CCDC126	PTDSS1	1.000
115	LOC147710	RLBP1	1.000
116	LOC644787	LOC643619	1.000
117	ICAM2	TCP11	1.000
118	UGT2B11	FBXW4	1.000
119	LOC652448	DENND2A	1.000
120	OR51M1	LOC642962	1.000
121	LOC645992	LOC389435	1.000
122	KIAA1713	NANOS3	1.000
123	IL1RAPL2	ARL17P1	1.000
124	WDR13	LOC643363	1.000
125	LOC387885	MARVELD1	1.000
126	CXorf21	LOC728797	1.000
127	MRPS27	ISOC2	1.000
128	LOC643640	PEX13	1.000
129	ZDHHC15	LOC645158	1.000
130	NT5C3	RHBDL2	1.000
131	LOC653061	PSCD2	1.000
132	PLK3	DGKG	1.000
133	ZNHIT1	ZNF239	1.000
134	SEC23A	SIGLEC10	1.000
135	KRTAP4-12	LOC728518	1.000
136	LOC642160	R3HDML	1.000
137	LOC652599	WDR51B	1.000
138	PAX6	LOC645253	1.000
139	HSD17B4	FLJ37228	1.000
140	WNT3	SPCS2	1.000
141	LOC651353	WBP2NL	1.000
142	C11orf74	DKFZp779B1634	1.000
143	HDDC2	STK33	1.000
144	MUC15	LOC646312	1.000
145	PCDHB6	C6orf142	1.000
146	LOC402682	RGS17	1.000
147	DUS4L	PDZRN4	1.000
148	LOC727987	HDC	1.000
149	JSRP1	RAP2C	1.000
150	SLC16A13	FBXO47	1.000
151	LOC148137	CLCN5	1.000
152	C9orf156	GNRH1	1.000
153	C1orf152	UBN1	1.000
154	RRAGA	CORO7	1.000
155	LOC392617	SCN1A	1.000
156	XRCC5	LOC648342	1.000
157	DKFZp434K1815	HP1BP3	1.000
158	LOC652730	SLC25A46	1.000
159	PSMB4	RAB14	1.000
160	FAM66E	PSMA2	1.000
161	ADCK2	76P	1.000
162	LOC645914	CYBRD1	1.000
163	LOC648060	BCLAF1	1.000
164	MAPRE1	PNPLA2	1.000
165	MAPRE1	EGFL9	1.000
166	COPE	TRAF1	1.000
167	LOC645789	LOC92312	1.000
168	MYO10	PGRMC1	1.000
169	LRP5	LOC653514	1.000
170	LOC646626	MORF4L2	1.000
171	ANKRD49	TMEM173	1.000
172	ANKRD49	SRD5A2L	1.000
173	DDEFL1	C14orf103	1.000
174	APOL6	IMMP1L	1.000

Table 4: interactions among the regulators and the target genes of the inferred GRN with top scores. (Contd..)

	TF Gene	Target Gene	Score
175	FLJ39501	TMEM132D	1.000
176	C11orf51	LOC643872	1.000
177	MRPL47	LOC654106	1.000
178	LOC653816	ATP5H	1.000
179	PP8961	DUSP8	1.000
180	TTC26	TAF1L	1.000
181	SRRM2	AGBL2	1.000
182	OR8H3	OSBPL9	1.000
183	PPIL6	APBA1	1.000
184	ALK	TRPC4	1.000
185	SLC6A3	LOC652840	1.000
186	PCDHB17	LOC442519	1.000
187	RHBDF1	PRDM13	1.000
188	LOC647315	B4GALT3	1.000
189	NLRC3	LIPG	1.000
190	FBXL16	RAPGEF4	1.000
191	IFI27	TCN2	1.000
192	H-plk	RAB18	1.000
193	ZNF557	PRR10	1.000
194	NME4	FANCF	1.000
195	MGC10433	LOC648170	1.000
196	MGC10433	LOC344065	1.000
197	PTPRN2	C20orf116	1.000
198	ECM2	NUDT11	1.000
199	N6AMT2	LOC649948	1.000
200	EID1	ATP10D	1.000
201	OR4D11	LOC642678	1.000
202	LOC730316	IBTK	1.000
203	LOC646129	RHBDD2	1.000
204	CHMP2B	S100A11	1.000
205	CYB561D2	MCHR1	1.000
206	TRIM32	RB1	1.000
207	LOC649937	ABC1	1.000
208	ENTPD1	TMCO5	1.000
209	C15orf15	UBE2V2	1.000
210	TPSD1	RFPL2	1.000
211	EIF5A	ULK3	1.000
212	C6orf118	SIVA1	1.000
213	LOC643948	EID2B	1.000
214	CCDC85A	HIP1	1.000
215	DCAL1	DDI1	1.000
216	LOC652190	LRRC17	1.000
217	LOC652575	AKAP13	1.000
218	LOC643089	LOC606495	1.000
219	PTF1A	CDYL2	1.000
220	ZNF701	MGC11257	1.000
221	LOC646331	LOC255313	1.000
222	LOC642277	ATAD4	1.000
223	SH3D19	LOC440737	1.000
224	FLJ35848	LOC647083	1.000
225	CAPN12	MAS1L	1.000
226	CA13	SPINK7	1.000
227	LOC646473	CRLS1	1.000
228	C1orf194	LOC653877	1.000
229	LOC344967	LOC643918	1.000
230	LOC644195	IPO7	1.000
231	LOC644195	C8orf31	1.000
232	ARR3	KIAA1344	1.000
233	SNORD42A	YIPF4	1.000
234	CENTG2	PPP1R14D	1.000
235	ZNF418	CCR10	1.000
236	LOC645836	SHANK1	1.000
237	LYZ	MGC25181	1.000
238	MGC10997	LOC283129	1.000
239	ANKRA2	CHRFAM7A	1.000
240	PPIL3	MGC5139	1.000
241	P2RY2	RASSF8	1.000
242	SMA5	LOC254398	1.000
243	C21orf33	CHD5	1.000
244	IL1R2	CEACAM4	1.000
245	C11orf17	GSG1L	1.000
246	APRIN	LOC727922	1.000
247	ALOX5	SIM1	1.000
248	SCAND1	DSP	1.000
249	LOC653145	CD200	1.000
250	PAK4	RSHL3	1.000
251	LOC729559	GPR128	1.000
252	POP5	HSPC142	1.000
253	METRNL	NXPH3	1.000
254	CYP4Z1	PTS	1.000
255	LOC649270	TAS2R1	1.000
256	BACH1	LOC644666	1.000
257	ANKRD30A	NIPA1	1.000
258	DYNC2LI1	NONO	1.000
259	KIAA1160	CT47.7	1.000
260	NAV1	LOC646845	1.000
261	DAG1	LOC283440	1.000
262	CSAG1	ACCN1	1.000

Table 4: interactions among the regulators and the target genes of the inferred GRN with top scores. (Contd..)

	TF Gene	Target Gene	Score
263	HK1	LOC650620	1.000
264	HK1	LOC642680	1.000
265	AMFR	SMC4	1.000
266	CTRB2	NSFL1C	1.000
267	FSTL4	KPNA6	1.000
268	TM2D3	A1BG	1.000
269	C22orf9	KIAA1257	1.000
270	LOC283152	LOC652472	1.000
271	NFASC	RNASEN	1.000
272	MRPL39	LOC645837	1.000
273	GGT1	GUCY1A3	1.000
274	CCNE2	RFC3	1.000
275	OTP	FLJ33708	1.000
276	CCNC	MRS2L	1.000
277	RBM9	RPL34	1.000
278	C4orf18	ZNF345	1.000
279	PCDHAC1	CUBN	1.000
280	DTNA	ZMYND19	1.000
281	ETV5	ARHGAP18	1.000
282	ITGBL1	QRICH1	0.623
283	SYNC1	KLHL9	0.589
284	PEX5	SCRN3	0.577
285	LOC652049	LOC401442	0.574
286	COL1A1	CLMN	0.563
287	TNFRSF10D	CLDN11	0.559
288	SETD2	NKG7	0.557
289	MICALCL	NODAL	0.555
290	CD302	PLRG1	0.553
291	ARL3	TMCC2	0.551
292	ZNF668	NUDCD1	0.550
293	LOC651296	RARA	0.548
294	BHLHB5	LOC643011	0.548
295	PCDHB2	RARS	0.544
296	ING4	C6orf162	0.543
297	LOC643729	STAT6	0.542
298	LOC645418	C8orf33	0.542
299	LOC554226	HSMPP8	0.542
300	KRT12	OR5J2	0.542
301	CAPN13	ATP6V1F	0.541
302	JAZF1	ACMSD	0.540
303	SYNGR4	LOC644652	0.538
304	YWHAZ	SLC16A11	0.536
305	HLA-DRB1	ALPL	0.535
306	KLHL24	LOC644408	0.534

	TF Gene	Target Gene	Score
307	THAP2	FXR2	0.534
308	UBE1L2	LOC644120	0.533
309	ZBTB22	LOC649174	0.532
310	INPP5A	MGC34824	0.532
311	METRN	DEFB114	0.531
312	TOMM22	LOC440389	0.530
313	SIGLEC9	LOC647778	0.529
314	LOC441775	UBE2B	0.528
315	LOC643879	LOC649009	0.527
316	ADAMTS10	PID1	0.527
317	MYBPHL	FBXO31	0.527
318	C16orf45	WDR42B	0.525
319	TMEM169	C14orf50	0.525
320	LOC646445	SEMA3F	0.525
321	BAP1	AKAP7	0.524
322	SLC2A12	ZNF180	0.522
323	LOC730272	AICDA	0.522
324	C11orf10	ARGFXP2	0.522
325	C7orf25	CPLX3	0.521
326	ZNF597	PTPRH	0.521
327	LOC644972	RPS6KA2	0.521
328	ASCL1	IFNA6	0.520
329	GPR119	DCP1A	0.520
330	CHGA	LOC646283	0.519
331	LAP3	LOC400965	0.519
332	LOC653663	IIP45	0.519
333	FIBP	ALG10	0.519
334	LOC651166	LOC652438	0.519
335	TMEM16F	CNN1	0.518
336	LTB4R	LOC652667	0.518
337	LOC650349	ARMCX1	0.517
338	LOC441775	COL19A1	0.517
339	SLC22A5	OCIAD2	0.516
340	ANGPTL5	LOC642460	0.516
341	HELT	NLGN4X	0.516
342	LOC643634	RHPN1	0.516
343	UBR1	UXS1	0.515
344	LOC440268	OR5P3	0.515
345	LOC652664	IFI35	0.515
346	USP10	ARL4A	0.515
347	ITFG3	TRIM2	0.515
348	PNPLA5	HCRT	0.514
349	PLA2G4D	C11orf31	0.514
350	HS2ST1	LOC401068	0.514

Table 4: interactions among the regulators and the target genes of the inferred GRN with top scores. (Contd..)

	TF Gene	Target Gene	Score
351	LOC645879	LOC642780	0.514
352	LOC643621	ANAPC1	0.513
353	PAX5	ARL2	0.513
354	GSTZ1	LOC646149	0.513
355	SSR2	GEMIN5	0.513
356	CSDE1	LOC653093	0.513
357	LOC645196	TMEM112B	0.512
358	LOC649094	KIF1A	0.512
359	USP10	RGR	0.512
360	KRTAP1-5	LOC642616	0.511
361	FLJ21839	LOC648744	0.511
362	SNAI1	CLNS1A	0.511
363	ZNF420	FEZ1	0.510
364	SELL	C17orf45	0.510
365	PTDSS1	GOT1	0.510
366	PDE4B	FLJ31945	0.509
367	LOC440359	NDUFA9	0.509
368	HPSE	XAB2	0.509
369	SLC36A2	SLC9A6	0.509
370	CCDC124	PLD2	0.509
371	C9orf45	LPIN3	0.509
372	SELPLG	TSPAN6	0.509
373	RHOH	AFF1	0.509
374	LOC647096	CMAS	0.508
375	FNBP1	FZR1	0.508
376	NOXA1	LOC642773	0.508
377	GPR143	FLJ16641	0.508
378	LOC644168	LOC652670	0.508
379	LOC644695	SUPT3H	0.508
380	LOC650058	PSMA5	0.507
381	LOC642198	ACY1L2	0.507
382	LOC144097	RAD54B	0.507
383	OR4P4	VDAC2	0.507
384	PROKR2	RAD17	0.507
385	ASB14	STIM2	0.507
386	LOC441996	SNIP	0.506
387	LOC151121	TKTL1	0.506
388	LOC653574	LOC729414	0.506
389	FLJ46358	PART1	0.506
390	DLG5	C21orf56	0.506
391	PXN	FN1	0.505
392	LOC652704	LOC653891	0.505
393	CSNK2B	HOXA2	0.505
394	MPP2	C10orf91	0.505
395	RPL34	FAM54B	0.505
396	CTNNA1	LOC644285	0.505
397	SOX9	CT47.6	0.505
398	LOC642702	KRT25	0.505
399	C6orf122	LOC643109	0.504
400	ERAS	LGALS13	0.504
401	LOC441179	FTHL17	0.504
402	FLJ45530	MTF2	0.504
403	FZD9	RNF152	0.504
404	PRKACG	MGC3196	0.504
405	LOC646774	LOC649317	0.504
406	CEACAM19	FAM98B	0.504
407	FAM62A	MGC15523	0.504
408	LOC651454	LOC442049	0.503
409	MUC13	LOC644748	0.503
410	PDZD8	EN2	0.503
411	FZD9	LOC388885	0.503
412	LOC402571	GJA5	0.503
413	ZNF468	GUCA1B	0.503
414	PRKAA1	LOC90835	0.503
415	ANKRD29	ZNF541	0.503
416	PFKP	MPPED2	0.502
417	RGS8	CLEC4A	0.502
418	RC3H2	LOC644187	0.502
419	KIAA0367	ZNF659	0.502
420	UBL4B	LOC728778	0.502
421	HDGFRP3	OSBPL3	0.502
422	LOC389517	GPR17	0.502
423	TAF1L	ERVWE1	0.502
424	LOC646568	LPIN2	0.502
425	LOC644986	LOC646010	0.502
426	STOM	SLC25A35	0.501
427	PHACTR4	ABCC1	0.501
428	LOC649260	PNMA1	0.501
429	C10orf113	PPTC7	0.501
430	TSPAN15	SLC7A11	0.501
431	BHLHB2	LOC651619	0.501
432	TINAGL1	ELAC1	0.501
433	PRNP	LOC441368	0.501
434	LOC642212	TNRC6C	0.501
435	FLJ39739	KIAA1712	0.501
436	FOXC2	IMPA2	0.501
437	ING2	TSPAN11	0.500
438	LOC652147	SRGN	0.500

Table 4: interactions among the regulators and the target genes of the inferred GRN with top scores. (Contd..)

	TF Gene	Target Gene	Score
439	C19orf39	APPL2	0.500
440	FLJ25439	LSM1	0.500
441	C14orf174	TADA2L	0.500
442	LOC131873	LOC651581	0.500
443	REEP5	FAS	0.500
444	ENTPD8	DDX41	0.500
445	SENP6	RNMTL1	0.500
446	RPL32	UCHL1	0.500
447	C1orf2	LOC196993	0.500
448	LEREPO4	LOC196993	0.500
449	CACNG8	UCHL1	0.500
450	ZKSCAN2	RNMTL1	0.500
451	LOC650678	DDX41	0.500
452	C19orf42	FAS	0.500
453	LOC652451	LOC651581	0.500
454	LOC642573	TADA2L	0.500
455	VANGL1	LSM1	0.500
456	PFAAP5	APPL2	0.500
457	LOC651511	SRGN	0.500
458	PRAMEF2	TSPAN11	0.500
459	PLAUR	IMPA2	0.499
460	BMP15	KIAA1712	0.499
461	LOC647416	TNRC6C	0.499
462	NFKBIL2	LOC441368	0.499
463	HIST1H2BK	ELAC1	0.499
464	ENDOGL1	LOC651619	0.499
465	SYNE2	SLC7A11	0.499
466	C3orf18	PPTC7	0.499
467	LOC643479	PNMA1	0.499
468	GRB10	ABCC1	0.499
469	SPRR2F	SLC25A35	0.499
470	BACE2	LOC646010	0.498
471	GTF3A	LPIN2	0.498
472	ABCG2	ERVWE1	0.498
473	IL17RA	GPR17	0.498
474	SHROOM1	OSBPL3	0.498
475	LOC132706	LOC728778	0.498
476	LOC646631	ZNF659	0.498
477	C6orf107	LOC644187	0.498
478	HSPC049	CLEC4A	0.498
479	TEKT4	MPPED2	0.498
480	SLC6A3	ZNF541	0.497
481	ZNF468	LOC90835	0.497

	TF Gene	Target Gene	Score
482	FLJ16331	GUCA1B	0.497
483	LOC647246	GJA5	0.497
484	LOC732192	LOC388885	0.497
485	GLIS1	EN2	0.497
486	ARF1	LOC644748	0.497
487	LOC652148	LOC442049	0.497
488	SAPS3	MGC15523	0.496
489	LOC653884	FAM98B	0.496
490	LOC643035	LOC649317	0.496
491	RSPO3	MGC3196	0.496
492	DEPDC6	RNF152	0.496
493	LOC652889	MTF2	0.496
494	IGFL2	FTHL17	0.496
495	SPRY4	LGALS13	0.496
496	LOC388692	LOC643109	0.496
497	LOC339782	KRT25	0.495
498	GRLF1	CT47.6	0.495
499	MYRIP	LOC644285	0.495
500	C17orf56	FAM54B	0.495
501	PIH1D2	C10orf91	0.495
502	PTPRQ	HOXA2	0.495
503	OR51B4	LOC653891	0.495
504	LOC729744	FN1	0.495
505	TMEM178	C21orf56	0.494
506	ENTPD4	PART1	0.494
507	OR52E8	LOC729414	0.494
508	SLURP1	TKTL1	0.494
509	PWWP2	SNIP	0.494
510	LOC644484	STIM2	0.493
511	CCNG2	RAD17	0.493
512	TXNL6	VDAC2	0.493
513	GJB6	RAD54B	0.493
514	HMGCR	ACY1L2	0.493
515	ZNF253	PSMA5	0.493
516	HSF2	SUPT3H	0.492
517	C9orf31	LOC652670	0.492
518	LOC641982	FLJ16641	0.492
519	C14orf177	LOC642773	0.492
520	LOC647131	FZR1	0.492
521	MGEA5	CMAS	0.492
522	TRIM39	AFF1	0.491
523	MNS1	TSPAN6	0.491
524	LRRC18	LPIN3	0.491

Table 4: interactions among the regulators and the target genes of the inferred GRN with top scores. (Contd..)

	TF Gene	Target Gene	Score
525	HHATL	PLD2	0.491
526	PLEKHF2	SLC9A6	0.491
527	ZMYND19	XAB2	0.491
528	LOC139735	NDUFA9	0.491
529	RAF1	FLJ31945	0.491
530	MKKS	GOT1	0.490
531	LOC649103	C17orf45	0.490
532	LOC645355	FEZ1	0.490
533	RAET1E	CLNS1A	0.489
534	MAFA	LOC648744	0.489
535	LOC400197	LOC642616	0.489
536	LOC652441	RGR	0.488
537	LOC649075	KIF1A	0.488
538	PPIL4	TMEM112B	0.488
539	LMO4	LOC653093	0.487
540	CACNA1B	GEMIN5	0.487
541	TM9SF3	LOC646149	0.487
542	HIST1H1T	ARL2	0.487
543	KPNA2	ANAPC1	0.487
544	MGC40499	LOC642780	0.486
545	GLT8D3	LOC401068	0.486
546	CD6	C11orf31	0.486
547	LOC653596	HCRT	0.486
548	MYD88	TRIM2	0.485
549	TUBA1A	ARL4A	0.485
550	LOC650174	IFI35	0.485
551	C18orf2	OR5P3	0.485
552	LOC376693	UXS1	0.485
553	C2orf34	RHPN1	0.484
554	CYP2A7	NLGN4X	0.484
555	LOC643346	LOC642460	0.484
556	MTNR1A	OCIAD2	0.484
557	XRN2	COL19A1	0.483
558	LOC647089	ARMCX1	0.483
559	RFC3	LOC652667	0.482
560	BBS10	CNN1	0.482
561	LOC653326	LOC652438	0.481
562	MRPL54	ALG10	0.481
563	HLA-DPA1	IIP45	0.481
564	RLN3	LOC400965	0.481
565	LOC642196	LOC646283	0.481
566	LOC645445	DCP1A	0.480
567	C19orf30	IFNA6	0.480

	TF Gene	Target Gene	Score
568	MAG1	RPS6KA2	0.479
569	RPL12	PTPRH	0.479
570	IFT140	CPLX3	0.479
571	KIAA1632	ARGFXP2	0.478
572	KIAA1840	AICDA	0.478
573	TUFM	ZNF180	0.478
574	FAM57A	AKAP7	0.476
575	LIPJ	SEMA3F	0.475
576	TCFL5	C14orf50	0.475
577	CDKN2C	WDR42B	0.475
578	WDR55	FBXO31	0.473
579	DEPDC2	PID1	0.473
580	OR2A12	LOC649009	0.473
581	LGALS8	UBE2B	0.472
582	LOC646906	LOC647778	0.471
583	NFATC4	LOC440389	0.470
584	GNAT2	DEFB114	0.469
585	IPO8	MGC34824	0.468
586	LOC653264	LOC649174	0.468
587	LOC644876	LOC644120	0.467
588	RWDD1	FXR2	0.466
589	STOX2	LOC644408	0.466
590	PSMD5	ALPL	0.465
591	LOC652226	SLC16A11	0.464
592	EXDL2	LOC644652	0.462
593	LDB2	ACMSD	0.460
594	LOC441268	ATP6V1F	0.459
595	LOC648603	OR5J2	0.458
596	C1orf141	HSMPP8	0.458
597	LOC442316	C8orf33	0.458
598	FLJ23152	STAT6	0.458
599	C1S	C6orf162	0.457
600	ZNF669	RARS	0.456
601	ZNF793	LOC643011	0.452
602	LOC651031	RARA	0.452
603	FLJ23049	NUDCD1	0.450
604	LOC653136	TMCC2	0.449
605	LOC643502	PLRG1	0.447
606	FOXH1	NODAL	0.445
607	SYTL2	NKG7	0.443
608	NME3	CLDN11	0.441
609	LOC652173	CLMN	0.437
610	PPP1R14B	LOC401442	0.426

Table 4: interactions among the regulators and the target genes of the inferred GRN with top scores. (Contd..)

	TF Gene	Target Gene	Score
611	LOC339745	SCRN3	0.423
612	LARP2	KLHL9	0.411
613	KIR3DL3	RPS21	0.411
614	LOC643731	METTL7A	0.394
615	FLJ90650	LOC203510	0.390
616	OR2B11	FLJ21839	0.389
617	LOC653764	KIAA1009	0.380
618	GPT	QRICH1	0.377
619	DPP3	TLOC1	0.377
620	TSHZ3	MANSC1	0.377
621	ITGB1BP2	SPINK5L2	0.375
622	UCHL1	LOC645811	0.375
623	C6orf52	MSH2	0.370
624	UTS2D	LOC441177	0.366
625	LOC283849	LOC645927	0.365
626	ABCD1	ACSL4	0.363
627	STELLAR	MAP2K1	0.362
628	TREML2	MTHFS	0.361
629	SMARCA2	LOC441907	0.360
630	CRELD1	C1orf142	0.358
631	PCDHGA5	LYRM5	0.358
632	IRF2	OR52E2	0.358
633	UGT1A6	LOC647819	0.358
634	ACTL7B	ZNF781	0.357
635	ENTPD3	PPP1R16A	0.357
636	LOC653800	CDKL3	0.357
637	SH3BGRL	EIF3S10	0.356
638	LOC647869	OR52E2	0.356
639	ASB11	XRCC2	0.356
640	OR52H1	T1560	0.356
641	LSM1	C6orf72	0.355
642	SUCLG1	OVCH2	0.355
643	ATOH8	LOC644001	0.354
644	WIBG	LAPTM5	0.354
645	ATP5L	ANKRD28	0.354
646	HMG20B	LOC644001	0.353
647	TSGA13	PLXNA3	0.353
648	LOC648000	GSTM2	0.353
649	MRPL34	RTP1	0.353
650	SLC25A37	CNTNAP3B	0.353
651	SPON2	NTRK1	0.352
652	TUSC1	ZNF580	0.352
653	HMOX1	WWP1	0.352
654	PFAAP5	KIAA0753	0.351

	TF Gene	Target Gene	Score
655	C19orf39	KIAA0753	0.351
656	LOC642568	PLEKHA6	0.351
657	ANGPTL2	GIMAP4	0.351
658	LOC728032	SLC5A1	0.351
659	LINGO2	ATP5S	0.351
660	CEP70	ACSL4	0.351
661	RPS27	LOC644668	0.350
662	HSD3B1	DMRTB1	0.350
663	LOC652796	PPP1R16A	0.350
664	CHCHD6	LOC642895	0.349
665	KPNA1	KRT15	0.349
666	COTL1	LOC642895	0.349
667	TMCO3	OR6B2	0.349
668	SNAPC5	LOC647711	0.348
669	ZNF474	AP1GBP1	0.348
670	VPS33A	MAP1LC3B	0.348
671	LOC644598	LCOR	0.348
672	DKFZp434I1020	OR2B6	0.348
673	SYDE2	NFAT5	0.347
674	LOC644925	ATP5S	0.347
675	LOC646756	LOC651166	0.347
676	TBK1	AHSG	0.347
677	LOC732402	C21orf128	0.346
678	POLDIP3	SHC1	0.346
679	ANKRD12	RTP1	0.346
680	TRIM13	MMP8	0.346
681	AKT3	FH	0.346
682	TMEM4	AP1GBP1	0.346
683	WDR57	KIAA1614	0.346
684	BDNF	RBPJ	0.346
685	AGTR1	LOC646300	0.345
686	LOC647726	LOC641518	0.345
687	ZDHHC15	LOC390205	0.345
688	ZNF582	ETFB	0.344
689	ZNF77	SRD5A1	0.344
690	LRRC47	PPIL4	0.344
691	MRPS5	NFXL1	0.344
692	ARVCF	MAP1LC3B	0.344
693	IL18	PACSIN1	0.344
694	C8orf58	ACBD4	0.344
695	KLF16	OR51S1	0.344
696	C8orf22	TMEM105	0.343
697	DEADC1	LOC644046	0.343
698	WDR23	LOC646507	0.343

Table 4: interactions among the regulators and the target genes of the inferred GRN with top scores. (Contd..)

	TF Gene	Target Gene	Score
699	ALOX5	KRT73	0.343
700	LOC139363	TRIM32	0.343
701	YARS2	LOC653154	0.343
702	LOC642132	RAB6B	0.342
703	CYP2C8	LOC653247	0.342
704	SOX5	LOC653113	0.342
705	OR6K2	AHSG	0.342
706	CSNK2A1P	C6orf146	0.342
707	LOC648256	LOC643283	0.341
708	KIAA0226	HS1BP3	0.341
709	LOC647046	FLJ41766	0.341
710	BCL2L2	C6orf72	0.341
711	L3MBTL4	NUP88	0.341
712	LOC150759	NY-REN-7	0.341
713	OR2M1P	LOC644668	0.341
714	C6orf130	PIP5K2B	0.340
715	NBEAL2	SSPN	0.340
716	LOC650846	LOC642820	0.340
717	RAD54L	FLJ41766	0.340
718	SLC25A30	SOAT2	0.340
719	IPMK	LOC642902	0.340
720	ZC3HC1	KIAA1614	0.340
721	TCEAL1	FLJ31813	0.340
722	LOC644468	ACBD4	0.339
723	PF4V1	MMP8	0.339
724	MLCK	NDP	0.339
725	KRIT1	RBPJ	0.339
726	C20orf166	NGDN	0.339
727	SOX9	HERC2	0.339
728	GLIS2	NGDN	0.339
729	LOC651747	C6orf49	0.339
730	LOC401442	DNHD1	0.339
731	EML2	EIF3S10	0.339
732	PTPRCAP	RAB6B	0.339
733	SIAE	LOC642623	0.339
734	BRD9	TBC1D25	0.339
735	SULT1A1	LOC650520	0.338
736	GAPDHS	RNH1	0.338
737	C10orf85	ZNF624	0.338
738	TCF12	C20orf94	0.338
739	PILRB	GPR50	0.338
740	NDUFV1	TPO	0.338
741	LOC644976	NFXL1	0.338
742	SPP2	OR6C1	0.338
743	FLJ32065	SSPN	0.338
744	ZNF354A	NAP1L2	0.338
745	LOC649905	C6orf146	0.338
746	PICK1	FOXH1	0.338
747	BTN3A2	LOC642902	0.338
748	FGF22	HERC2	0.338
749	DCP1B	ZNF624	0.338
750	MAP1A	RP2	0.338
751	KIAA1462	SDHD	0.337
752	DSE	WDR6	0.337
753	CHCHD3	NY-REN-7	0.337
754	LOC653696	SGEF	0.337
755	ARR3	DNHD1	0.337
756	LOC644168	TPO	0.337
757	HAND1	SDHD	0.337
758	LOC643035	FLJ31813	0.337
759	LOC113230	GKAP1	0.336
760	ASH2L	CDKL3	0.336
761	KIAA0040	MGC50722	0.336
762	ALK	LOC650874	0.336
763	TAF11	SYCP2	0.336
764	LOC653784	SHC1	0.336
765	RFESD	USP25	0.336
766	GPR78	LOC650520	0.336
767	FRAP1	ICMT	0.336
768	KLHL6	NUDT14	0.336
769	C16orf44	SGEF	0.336
770	PYGB	RPL10L	0.336
771	ESM1	GPR50	0.335
772	LOC652203	FIP1L1	0.335
773	CALML4	C20orf94	0.335
774	VDAC1	WDR68	0.335
775	ZNF511	LOC441191	0.335
776	LOC647911	NAP1L2	0.335
777	LOC90342	NUDT14	0.335
778	LAMP3	LOC644046	0.335
779	DLEU1	LOC441191	0.335
780	LOC389842	FOXH1	0.335
781	LOC652789	EPAS1	0.335
782	FLJ16641	USP25	0.335
783	SULT4A1	WDR68	0.335
784	LOC440820	LOC642648	0.335
785	PRIC285	PPIL4	0.335
786	EDG6	SUPT5H	0.335

Table 4: interactions among the regulators and the target genes of the inferred GRN with top scores. (Contd..)

	TF Gene	Target Gene	Score
787	C20orf50	NCAPD3	0.334
788	LOC729355	SYCP2	0.334
789	CHMP4A	LOC642623	0.334
790	LOC652416	WWP1	0.334
791	OR6B1	TTC27	0.334
792	NUPR1	TTC27	0.334
793	LOC648230	RNH1	0.334
794	LOC647760	RPL10L	0.334
795	SNORD4B	LOC642718	0.334
796	GEMIN7	LOC642718	0.334
797	LOC400236	TYSND1	0.334
798	AKAP8	TBC1D25	0.334
799	MAF1	NCAPD3	0.334
800	LYRM5	OR6C1	0.334
801	ARHGAP21	TYSND1	0.333
802	SYTL1	C6orf49	0.333
803	OR2AT4	SUPT5H	0.333
804	LOC647485	NUP88	0.333
805	C20orf106	RP2	0.333
806	EMR4	TYSND1	0.333
807	SAPS3	LOC651166	0.333
808	XAB1	PLXNA3	0.333
809	ZYG11B	LOC646300	0.333
810	GDA	LOC642648	0.333
811	LOC646665	LOC642648	0.333
812	NSDHL	EPAS1	0.333
813	LAMC3	EPAS1	0.332
814	AUP1	MGC50722	0.332
815	USF1	GKAP1	0.332
816	SNORD100	ICMT	0.332
817	TMEM61	LOC650874	0.332
818	ZNF620	FIP1L1	0.332
819	HNRPA2B1	LOC642718	0.332
820	ENDOGL1	SUPT5H	0.332
821	IFNA13	LOC645811	0.332
822	TBC1D25	FIP1L1	0.332
823	LOC643958	NCAPD3	0.332
824	dJ341D10.1	TTC27	0.332
825	GDF2	ICMT	0.332
826	LOC389203	WDR6	0.332
827	LOC649738	WDR6	0.332
828	FHL5	KRT15	0.332
829	LOC651763	NDP	0.332
830	DPP9	LOC642820	0.331

	TF Gene	Target Gene	Score
831	LOC220998	LOC650874	0.331
832	RAVER1	LOC653113	0.331
833	LOC51035	MGC50722	0.331
834	MRPL53	SOAT2	0.331
835	LOC642566	SLC5A1	0.331
836	LOC653829	GKAP1	0.331
837	SMARCA2	LOC441177	0.331
838	C9orf127	LOC653247	0.331
839	MST1R	RPL10L	0.331
840	ZNF750	NFAT5	0.330
841	LIPF	LOC641518	0.330
842	PPP4R1L	PACSIN1	0.330
843	LOC648976	SRD5A1	0.330
844	GALNT7	OVCH2	0.330
845	LOC642229	TMEM105	0.330
846	RNMTL1	LCOR	0.330
847	LOC645401	LOC653154	0.330
848	ZNF295	LOC643283	0.330
849	WDR64	WDR68	0.330
850	FLJ46363	LOC441191	0.330
851	TMEM147	PIP5K2B	0.330
852	KIAA1727	PIP5K2B	0.330
853	APRIN	XRCC2	0.330
854	PSMD2	LOC646507	0.330
855	LOC653326	HS1BP3	0.330
856	CCRK	RP2	0.330
857	LOC652156	SYCP2	0.329
858	GTF3C3	KRT73	0.329
859	H2AFB1	NUDT14	0.329
860	DHX16	GSTM2	0.329
861	SLC25A21	MAP2K1	0.329
862	TMEM4	T1560	0.329
863	LOC653338	HS1BP3	0.329
864	LOC343384	NDP	0.329
865	LOC651051	TRIM32	0.329
866	DEFB32	USP25	0.329
867	LOC646040	SOAT2	0.329
868	LOC654350	OR51S1	0.329
869	LOC652456	LOC643283	0.329
870	BIRC2	ZNF781	0.328
871	FLJ31222	LOC390205	0.328
872	LOC651648	ANKRD28	0.328
873	LOC642196	LOC642820	0.328
874	LOC653701	OR6C1	0.328

Table 4: interactions among the regulators and the target genes of the inferred GRN with top scores. (Contd..)

	TF Gene	Target Gene	Score
875	TMEM16J	TRIM32	0.328
876	C8orf34	OR6B2	0.328
877	EPS8	GIMAP4	0.328
878	TUBB4Q	PLEKHA6	0.328
879	OR2L2	FH	0.328
880	AIM1	OR2B6	0.328
881	LOC643783	ETFB	0.328
882	LOC389174	KRT73	0.328
883	BPI	C6orf49	0.328
884	DUSP26	ETFB	0.328
885	ZNF521	SGEF	0.328
886	LOC440607	TBC1D25	0.328
887	ZNF789	RNH1	0.328
888	GRK5	OR51S1	0.328
889	SFXN5	LOC646507	0.328
890	TDGF1	LOC653154	0.327
891	PRPS1L1	FOXH1	0.327
892	ZNF532	LOC390205	0.327
893	RAC1	LOC642623	0.327
894	LOC651029	LOC647819	0.327
895	EXOC3L2	LOC653247	0.327
896	LOC391356	NAP1L2	0.327
897	CCRL2	C21orf128	0.327
898	HMFN0672	C21orf128	0.327
899	LOC441018	TMEM105	0.327
900	RNUXA	LOC653113	0.327
901	SPON1	SDHD	0.326
902	PCDH21	FH	0.326
903	VIM	C20orf94	0.326
904	HS2ST1	LOC647711	0.326
905	CSDA	NUP88	0.326
906	KIAA1257	GPR50	0.326
907	CRBN	PACSIN1	0.326
908	LOC642602	DMRTB1	0.326
909	USF1	LOC647711	0.326
910	KLK2	LOC650520	0.326
911	ZNF34	ZNF580	0.326
912	76P	SRD5A1	0.325
913	LOC645683	TPO	0.325
914	LOC644038	LOC641518	0.325
915	VPS41	DMRTB1	0.325
916	LHFPL4	NTRK1	0.325
917	LOC646241	DNHD1	0.324

	TF Gene	Target Gene	Score
918	LOC642111	OR2B6	0.324
919	LOC648625	LYRM5	0.324
920	THRAP4	ZNF624	0.324
921	LOC643836	FLJ31813	0.324
922	C1orf84	CNTNAP3B	0.324
923	EFCBP1	CNTNAP3B	0.324
924	FLJ10986	OR6B2	0.323
925	SERPINA7	HERC2	0.323
926	LOC644787	NTRK1	0.323
927	NAV3	LAPTM5	0.323
928	VAMP3	LAPTM5	0.323
929	LOC644320	ZNF580	0.323
930	LOC643109	NY-REN-7	0.323
931	KIAA0565	METTL7A	0.322
932	LOC645172	LOC642902	0.322
933	C6orf174	NFAT5	0.322
934	LLGL2	MTHFS	0.322
935	RNF150	LCOR	0.322
936	RHBDD2	LOC644046	0.322
937	NEB	JAZF1	0.322
938	CIB1	LOC646300	0.322
939	COL4A5	SSPN	0.322
940	BTN3A1	NGDN	0.322
941	LOC339209	PPIL4	0.321
942	LOC646588	C1orf142	0.321
943	LOC339692	GIMAP4	0.321
944	HBB	C1orf142	0.321
945	LOC648981	PLEKHA6	0.321
946	CEBPG	LOC441907	0.321
947	LOC392145	LOC651166	0.320
948	ELK4	C6orf146	0.320
949	TMEM98	SPINK5L2	0.320
950	PLTP	KRT15	0.320
951	LOC652838	LOC645927	0.319
952	LPIN2	LOC441907	0.319
953	TTC27	FLJ41766	0.319
954	LOC642548	RAB6B	0.319
955	LOC644916	LYRM5	0.318
956	GLCCI1	GSTM2	0.318
957	DSCR9	SLC5A1	0.318
958	PTPRH	ANKRD28	0.318
959	LOC642168	NFXL1	0.318
960	MYO15A	SHC1	0.317

Table 4: interactions among the regulators and the target genes of the inferred GRN with top scores. (Contd..)

	TF Gene	Target Gene	Score
961	C14orf94	ACBD4	0.317
962	LOC649991	MTHFS	0.316
963	OR5BU1	SLC16A7	0.316
964	COPZ2	LOC645927	0.316
965	D4ST1	TLOC1	0.315
966	LOC731777	FLJ21839	0.315
967	SERPINB8	LOC647819	0.315
968	ALKBH4	MSH2	0.315
969	LOC642312	T1560	0.315
970	PORCN	OVCH2	0.315
971	GALR3	RBPJ	0.315
972	CCDC59	MSH2	0.315
973	SPATA2	MMP8	0.315
974	LOC728285	XRCC2	0.315
975	C3orf10	KIAA1614	0.315
976	LOC648622	ZNF781	0.314
977	TPD52L2	PLXNA3	0.314
978	LOC731076	WWP1	0.314
979	TSLP	LOC203510	0.314

	TF Gene	Target Gene	Score
980	STXBP5	MANSC1	0.313
981	LOC649067	AHSG	0.312
982	NUP210L	KIAA1009	0.311
983	LOC641694	MANSC1	0.310
984	ZNF274	MAP2K1	0.309
985	LOC641738	KIAA1009	0.309
986	GSTM3	LOC644668	0.309
987	C6orf85	MAP1LC3B	0.308
988	C9orf66	TLOC1	0.307
989	LOC648408	CDKL3	0.307
990	LOC732192	AP1GBP1	0.306
991	PHLPPL	EIF3S10	0.305
992	VPRBP	SPINK5L2	0.305
993	LOC727957	C6orf72	0.304
994	WIPF1	LOC441177	0.303
995	SLCO1B3	ATP5S	0.303
996	CYP2C18	LOC642895	0.302
997	U2AF1L4	RTP1	0.301
